# Investigating Rewards and Deposit Contract Financial Incentives for Physical Activity Behavior Change Using a Smartphone App: Randomized Controlled Trial

**DOI:** 10.2196/38339

**Published:** 2022-10-06

**Authors:** David R de Buisonjé, Thomas Reijnders, Talia R Cohen Rodrigues, Santhanam Prabhakaran, Tobias Kowatsch, Stefan A Lipman, Tammo H A Bijmolt, Linda D Breeman, Veronica R Janssen, Roderik A Kraaijenhagen, Hareld M C Kemps, Andrea W M Evers

**Affiliations:** 1 Health, Medical and Neuropsychology Unit Institute of Psychology Leiden University Leiden Netherlands; 2 Department of Human-Centered Design Faculty of Industrial Design Engineering Delft University of Technology Delft Netherlands; 3 Centre for Digital Health Interventions Department of Management, Technology, and Economics ETH Zurich Zurich Switzerland; 4 Institute for Implementation Science in Health Care University of Zurich Zurich Switzerland; 5 School of Medicine University of St. Gallen St. Gallen Switzerland; 6 Erasmus School of Health Policy & Management Erasmus University Rotterdam Rotterdam Netherlands; 7 Faculty of Economics and Business University of Groningen Groningen Netherlands; 8 Department of Cardiology Leiden University Medical Center Leiden Netherlands; 9 Hearts4People Foundation Amsterdam Netherlands; 10 Department of Cardiology Máxima Medical Center Veldhoven Netherlands; 11 Medical Delta Leiden University, Technical University Delft, Erasmus University Delft Netherlands

**Keywords:** eHealth, behavior change, rewards, reward learning, financial incentives, deposit contracts, commitment contracts, physical activity, mobile phone

## Abstract

**Background:**

Financial incentive interventions for improving physical activity have proven to be effective but costly. Deposit contracts (in which participants pledge their own money) could be an affordable alternative. In addition, deposit contracts may have superior effects by exploiting the power of loss aversion. Previous research has often operationalized deposit contracts through loss framing a financial reward (without requiring a deposit) to mimic the feelings of loss involved in a deposit contract.

**Objective:**

This study aimed to disentangle the effects of incurring actual losses (through self-funding a deposit contract) and loss framing. We investigated whether incentive conditions are more effective than a no-incentive control condition, whether deposit contracts have a lower uptake than financial rewards, whether deposit contracts are more effective than financial rewards, and whether loss frames are more effective than gain frames.

**Methods:**

Healthy participants (N=126) with an average age of 22.7 (SD 2.84) years participated in a 20-day physical activity intervention. They downloaded a smartphone app that provided them with a personalized physical activity goal and either required a €10 (at the time of writing: €1=US $0.98) deposit up front (which could be lost) or provided €10 as a reward, contingent on performance. Daily feedback on incentive earnings was provided and framed as either a loss or gain. We used a 2 (incentive type: deposit or reward) × 2 (feedback frame: gain or loss) between-subjects factorial design with a no-incentive control condition. Our primary outcome was the number of days participants achieved their goals. The uptake of the intervention was a secondary outcome.

**Results:**

Overall, financial incentive conditions (mean 13.10, SD 6.33 days goal achieved) had higher effectiveness than the control condition (mean 8.00, SD 5.65 days goal achieved; *P*=.002; *ηp^2^*=0.147). Deposit contracts had lower uptake (29/47, 62%) than rewards (50/50, 100%; *P*<.001; Cramer *V*=0.492). Furthermore, 2-way analysis of covariance showed that deposit contracts (mean 14.88, SD 6.40 days goal achieved) were not significantly more effective than rewards (mean 12.13, SD 6.17 days goal achieved; *P*=.17). Unexpectedly, loss frames (mean 10.50, SD 6.22 days goal achieved) were significantly less effective than gain frames (mean 14.67, SD 5.95 days goal achieved; *P*=.007; *ηp^2^*=0.155).

**Conclusions:**

Financial incentives help increase physical activity, but deposit contracts were not more effective than rewards. Although self-funded deposit contracts can be offered at low cost, low uptake is an important obstacle to large-scale implementation. Unexpectedly, loss framing was less effective than gain framing. Therefore, we urge further research on their boundary conditions before using loss-framed incentives in practice. Because of limited statistical power regarding some research questions, the results of this study should be interpreted with caution, and future work should be done to confirm these findings.

**Trial Registration:**

Open Science Framework Registries osf.io/34ygt; https://osf.io/34ygt

## Introduction

### Background

Since the beginning of time, humans have been developing tools and technologies that have made life easier. These technological advances have led to historically unprecedented levels of physical inactivity [1]. For example, currently, only 23% of adults in the United States meet the recommended guidelines for physical activity [2]. Although physical inactivity is linked to chronic disease and early death [3], increasing physical activity reduces the risk of chronic disease, has positive effects on mental health, and increases longevity [4]. Importantly, the positive effects of physical activity are observed not only for intense aerobic training but also for the mere number of steps taken in daily life [5,6]. Intervening on improving daily step counts has the advantage of being objectively measurable (compared with self-reports), low cost (compared with pharmaceutical treatment), and relatively easy to implement in daily life (compared with gym-based aerobic training), and as a result, it is also suitable for deprived, vulnerable, and older populations worldwide. Therefore, stimulating an increase in daily step counts appears to be a promising and feasible avenue to help humanity become healthier and happier and to live longer.

Although many people are aware of the benefits of physical activity and have positive intentions to be (more) physically active, achieving sufficient physical activity in daily life is not achieved by many [7]. The finding that positive intentions do not always translate into the desired behavior has been linked to the intention-behavior gap and has been found in a variety of (health) behaviors [8], including physical activity [7]. Insights from behavioral economics help explain the causes of the intention-behavior gap. A key finding is that people are present biased [9]. Present bias refers to the tendency of people to be more strongly driven by consequences in the here and now, rather than by the long-term consequences of their decisions. Consequently, people tend to procrastinate. Although differences among individuals exist, the general pattern found is one wherein “people grab immediate rewards and avoid immediate costs in a way our long-run selves do not appreciate” [10]. Present bias has been shown to apply to health behavior in general [11] and to physical activity specifically [12]. For example, people with a stronger present bias have lower levels of physical activity, arguably because they overweight the short-term and often negative consequences of physical activity (eg, increased heart rate and sweating) and assign a lower value (ie, discount) to the long-term positive consequences of physical activity (eg, longevity) [12]. Present bias, therefore, helps explain why despite having good intentions to achieve long-term health goals, people are prone to fall for immediate temptation.

Present bias also helps explain why introducing financial incentives might be suitable as an intervention strategy for health behavior change. Offering immediate financial incentives for healthy behavior takes advantage of the present bias by introducing a monetary benefit in the here and now. As such, people no longer have to *wait* for the delayed rewards of healthy behavior to emerge but instead are immediately rewarded. Indeed, meta-analyses and systematic reviews have shown that financial incentives are an effective tool for promoting (at least short-term) health behavior changes, such as improving diet [13], combating substance use [13], increasing physical activity [14,15], weight loss [13], smoking cessation [15,16], and increasing vaccination uptake [16]. Financial incentives are often added as a supplement to already active behavior change interventions, roughly doubling the odds of successful behavior change [15]. For physical activity, a recent meta-analysis (N=6074) on the effectiveness of financial incentives on step counts showed an average daily increase of approximately 600 steps (10%-15%) during active intervention [14].

Another relevant insight from behavioral economics is that people are loss averse [17]. This refers to the tendency of individuals to assign larger weight to potential losses associated with their behavior than to potential gains. Losses and gains are defined with respect to a reference point; for example, individuals’ current status quo, their expectations, or goals [17]. Loss aversion and reference points have been shown to be important in health-related decision-making [18] and might lead to suboptimal decision-making for physical activity if it causes people to outweigh what they might lose by being physically active (eg, time and energy) over what they might gain (eg, satisfaction after a workout). Furthermore, loss aversion is often used to motivate financial incentive designs that involve potential losses rather than rewards only [19,20], such as deposit contracts.

Deposit contracts are a specific form of financial incentive wherein people deposit their own money and can earn it back contingent on behavior change [21]. There are several real-world commercial products (eg, Waybetter [22] and Stickk [23]) with deposit contracts that have proven to be commercially viable and claim to help people change their behavior. While rewards involve the introduction of a pleasant stimulus to increase behavior (ie, positive reinforcement), deposit contracts involve the alleviation of an aversive stimulus (avoiding loss of money) to increase behavior (ie, negative reinforcement) [24]. Deposit contracts offer several advantages over reward-based incentives. First, although both rewards and deposit contracts bring an incentive into the present, a deposit contract brings a risk of loss into the present and thus should be more effective because it capitalizes on loss aversion [19]. Second, the use of reward-based financial incentives for physical activity imposes a significant cost (eg, approximately US $1.50 per day per person, see the study by Mitchell et al [14]), whereas the use of deposit contracts introduces (partial) cost sharing by recipients. Such cost sharing may be desirable, for example, to employers promoting physical activity among employees [25]. Moreover, while rewarding people for behavior that others perform without receiving rewards might be considered unfair, having people voluntarily deposit their own money avoids this ethical concern [26].

Existing evidence indicates that deposit contracts are effective in helping people lose weight [26], stop smoking [19,27], and increase physical activity [20,21,24,28-30]. However, the voluntary uptake of deposit contracts is generally low [19,31]. In fact, some authors suggest that those who would benefit the most from interventions using incentives with potential losses are not likely to enter into them [32,33]. However, comparing the evidence on the uptake and effectiveness of deposit contracts for physical activity among studies is complicated, as operationalizations differ substantially. In particular, 3 different types of deposit contracts can be distinguished. First, in line with their potential to promote cost sharing, several authors have used completely self-funded deposit contracts [31,34]. Without the potential for financial gain, such self-funded deposit contracts involve only losses compared with the status quo. Second, uptake of deposit contracts is often encouraged through “matching” individuals’ contribution into the deposit scheme or combining deposits with a reward-based incentive [19,35,36]. Such matched deposit contracts thus involve both potential gains and losses compared with the status quo. Third, some authors have used loss framing to mimic the feelings of loss involved in a deposit contract without actually requiring individuals to put their own money at risk [20,24]. For example, in a loss-framed condition, Patel et al [20] promised respondents US $42 up front of which they could then lose US $1.40 for every day they did not attain physical activity goals. This loss-framed condition proved more effective in promoting physical activity compared with a gain-framed condition in which respondents simply earned US $1.40 for every day they attained physical activity goals. However, participants in all conditions of this study faced no actual losses, but in fact were making gains compared with their preintervention status quo.

### This Study

In this study, we investigate the impact of deposit contracts on increasing physical activity by disentangling the effects of incurring actual losses (through self-funding) and loss framing. We will use an actual deposit contract (ie, a stick) that requires participants to make a deposit of their own money before the intervention starts and compare this with receiving a reward (ie, a carrot) of equivalent size. In line with the study by Adams et al [37], we refer to this as the *direction* of incentives. Furthermore, we will investigate whether loss framing (compared with gain framing) enhances the effectiveness of both reward and deposit contract incentives. First, we expect that, overall, incentive conditions are more effective than an active no-incentive control condition (H1). Second, we hypothesize that deposit contracts will have lower uptake than regular rewards (H2); however, deposit contracts are expected to be more effective than regular rewards for those that partake in the intervention (H3). In addition, we hypothesize that loss framing an incentive will increase effectiveness compared with gain framing (H4). Finally, we propose that incentives in which both direction of the incentive and framing of the incentive are loss congruent (ie, loss-framed deposit contracts) are most likely to invoke loss aversion and are therefore especially effective in promoting physical activity (H5).

## Methods

### Participants

We recruited healthy participants aged between 18 and 30 years through a university research participation system (SONA), flyers on campus, and posts on social media. Participants had to be willing to improve their physical activity, own a smartphone, and be proficient in English. A priori sample size calculations with G*Power [38] suggested a minimum sample size of 199 for detecting a between-conditions difference in effectiveness with a medium effect size (f=0.20), 80% power, and an α of .05 (analysis of covariance [ANCOVA] with 5 groups). On the basis of a similar research [39] that showed a relatively high dropout rate between recruitment and participation, we assumed a dropout rate of 20% and aimed to recruit 240 eligible participants. Participants were excluded if they reported any medical condition that could hinder their physical activity (based on their response to the Physical Activity Readiness Questionnaire) [40]. A detailed description of the flow of participants through the study, including reasons for exclusion and dropout, is provided in Multimedia Appendix 1. All the participants who completed the study had a chance to win 1 of 3 grand prizes (3 Fitbit devices worth €100 [at the time of writing: €1=US $0.98]) and 1 of 50 small prizes (50 webshop vouchers worth €10) in a raffle. Participants who were first-year psychology students at Leiden University additionally received research credits (needed to complete their first year).

### Ethics Approval

We obtained informed consent before the start of the study. This study was approved by the Psychology Research Ethics Committee of Leiden University (2020-02-24-T. Reijnders-V2-2089), and the study protocol was preregistered on the Open Science Framework [41].

### Materials

The intervention for this study was delivered entirely on the web via the Benefit Move app, which the participants downloaded on their smartphones. The Benefit Move app was implemented using MobileCoach [42,43], an open-source software platform for smartphone-based and chatbot-delivered behavioral interventions (eg, study by Kowatsch et al [44]) and ecological momentary assessments (eg, study by Tinschert et al [45]). MobileCoach was developed by the Centre for Digital Health Interventions at Eidgenössische Technische Hochschule Zürich and the University of St. Gallen in Switzerland [46]. The Benefit Move app had two main functions: (1) objectively measuring physical activity and (2) communicating with the participant.

**Figure 1:**
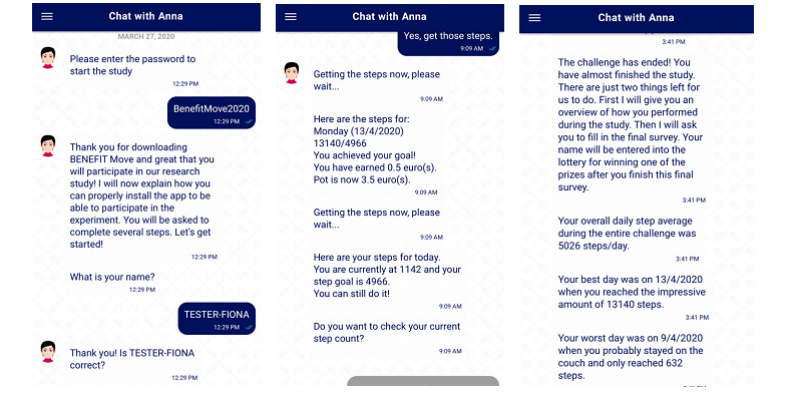
Impression of the Benefit Move app.

To measure physical activity, the Benefit Move app asked the participants for permission to retrieve step counts from existing health apps already installed on their smartphones. Most smartphones have a gyroscope-based pedometer or location-tracking device integrated to record movements made while the phone is being carried. Algorithms recode the raw data from these sensors into an estimated step count, which is then stored in the database of apps, such as Apple Health and Google Fit. Depending on the operating system, Benefit Move would pull data from either Google Fit [47] for Android or Apple’s Health Kit [48] for iOS. Overall, out of 126 participants, 67 (53.2%) used Apple iOS devices and 59 (46.8%) used Android devices. The percentage of Apple iOS users ranged from 41.1% to 69.6% across conditions and was considered to be spread evenly across conditions. Both of these apps showed good validity for measuring step counts [49,50]. The Benefit Move app retrieved these data to provide a tailored step goal at the start of the intervention and to record step counts during the intervention. During the intervention phase, at any given time, the participant could click a button to retrieve the up-to-date step count at that moment. In addition, to communicate with the participant, an automated digital coach (chatbot) sent daily prompts to provide the participant with feedback about goal progress, their accumulated financial earnings or losses, and a trigger to click the button for step count retrieval (Figure 1 provides an impression of the app).

### Measures

#### Baseline Survey

The baseline survey was administered during onboarding in the app to obtain basic demographic information such as sex, birth year, nationality, country of residency, education level, employment status, subjective estimation of income relative to peers, and subjective estimation of weight status (Multimedia Appendix 2 provides an overview of the survey items).

#### Final Survey

The final survey was administered after the intervention was completed. First, as a sensitivity check, we asked the participants whether they carried their smartphone with them more often because of the intervention (Multimedia Appendix 3 provides an overview of the final survey items). Furthermore, we asked the participants if they cheated the intervention but assured them that their answer would not impact the payout of incentives. We also performed a contamination check to explore whether participants were aware of the condition that others were assigned. Because the intervention coincided with the worldwide COVID-19 pandemic, we included several items to assess its impact on our study. First, we assessed whether participants experienced influenza-like symptoms, whether these symptoms led them to be less physically active, and, in general, whether they engaged in less physical activity owing to the COVID-19 pandemic. Furthermore, we administered the Generalized Anxiety Disorder-7 [51], a brief 7-item measure that assesses generalized anxiety symptoms that could be related to the COVID-19 pandemic. Finally, as a manipulation check, we included 2 items (answered on a 10-point Likert scale from 1=totally disagree to 10=totally agree) that asked whether participants experienced a feeling of loss during the intervention (“I felt that I was losing money if I did not increase my step count”) and whether they experienced goal commitment (“I felt strongly committed to the goal of increasing my step count”).

### Procedure

After recruitment, all the participants were put on a waitlist before they received the screening survey and informed consent. One week before the start of the intervention, participants completed the screening survey with the inclusion and exclusion criteria and provided digital informed consent. Thereafter, eligible participants received a URL to the iOS or Android app stores where they could download the Benefit Move intervention app and install it on their smartphone. Once the participants installed the app, they were asked to complete onboarding in the app within 2 days. Thereafter, participants were sent a link to the survey platform LimeSurvey that opened within the Benefit Move app. Here, they filled in the baseline survey (for more details, see the Baseline Survey section) and then returned to the app after completion. Participants were excluded from the study if they did not complete the onboarding process and baseline survey before the start of the intervention.

After participants completed the baseline survey, they received a tailored step goal based on their 7-day historic daily step average that was retrieved through Google Fit or Apple Health. Retrieving step counts for 7 consecutive days should accurately estimate habitual activity levels of individuals [52], and providing an individualized and realistic goal should increase intervention effectiveness [14]. A limitation to using a 7-day historic step count is that meteorological factors could impact baseline levels of activity [53]. If historic data were available, the participant was assigned a goal that was 120% of the historic daily step average. For example, someone who, in the 7 days before goal setting, took an average of 5000 steps per day would automatically receive a 6000 steps daily step goal. If no historic data were available, the participant was assigned a default step goal of 10,000 steps per day because it is an often-used guideline for sufficient physical activity [54].

All the participants started simultaneously with the 20-day intervention on Monday, March 30, 2020, at 9 AM. Owing to the COVID-19 pandemic, a partial lockdown was issued by the Dutch government on March 15, 2020. Onboarding for this study (and retrieval of 7 days of historic step counts) was performed from March 23, 2020, until the active study phase started on March 30, 2020. Therefore, it is possible that the estimates of the baseline activity were lower than normal. Each day during the 20-day intervention, the participants received a push notification at 9 AM. This notification prompted them to click a button to retrieve their step count performance of the previous day and get an update on the progress for the current day. If the user skipped doing this for several days but then responded and requested an update, the feedback for multiple days was given in separate consecutive messages, with a separate update message per day. The feedback per day consisted of the achieved step count compared with the daily step goal, a conclusion about whether the goal was achieved or not, the money that was earned or lost on that day, and the running total of earnings or losses during the entire intervention (Figure 1 provides an example). On the basis of their study conditions, participants received different instructions at the start of the intervention and received different feedback messages during the intervention.

### Study Conditions

We used a 2 incentive direction (reward or deposit) × 2 feedback frame (gain or loss) design with an additional control condition. The participants were automatically randomized to these 5 conditions by the app.

#### Condition 1: Control Condition

Participants received an active basic intervention with a tailored goal and daily feedback on their goal progress without a financial incentive or specific framing of feedback.

#### Condition 2: Reward and Gain Frame Condition

After having been assigned their step goal, participants were informed that they would receive a monetary reward of a maximum of €10 for achieving their step goals during the intervention (the incentive amount of €10 was determined in a pilot study during which we sent a short survey to 26 students to assess what incentive amount they would find stimulating and acceptable). More specifically, to create a gain frame, they were informed that there was an empty pot at the start of the intervention and that for every successful goal achievement, they would receive €0.50 that would be added to the pot. If they were not successful, nothing would be added to the pot. After their condition was explained to the participants, we explicitly asked them if they wanted to participate in this challenge (this is especially relevant for participants in the deposit conditions, as they will be asked to make a monetary payment to the experiment). After they explicitly agreed to the specific challenge that was presented to them, the participants were instructed to wait until the intervention started the next Monday morning.

#### Condition 3: Reward and Loss Frame Condition

After having been assigned their step goal, participants were also informed that they would receive a monetary reward of a maximum of €10 for achieving their step goals during the intervention. However, to create a loss frame, and in contrast to the gain frame condition, they were informed that there was a full pot with €10 at the start of the intervention and that for every goal failure €0.50 would be deducted from the pot. If they were successful, nothing would be deducted from the pot.

#### Condition 4: Deposit and Gain Frame Condition

After having been assigned their step goal, participants in the deposit and gain frame condition were asked to deposit €10 of their own money via bank transfer to improve their commitment to the challenge. In all cases, the full amount was refunded after the intervention, but participants were unaware of this and were informed that the amount they would get back would depend on their performance during the intervention. More specifically, they were informed that there was an empty pot at the start of the intervention and that for every successful goal achievement, €0.50 would be added to the pot. If they were not successful, nothing would be added to the pot. The final amount of the pot would be the amount of their deposit that would be returned to them after the intervention.

After their condition was explained to them, we explicitly asked the participants if they wanted to participate in this challenge. When participants agreed to participate, they were sent a digital payment request via “Tikkie” (a direct digital payment URL) in the app. By clicking on this payment request, they directly transferred €10 of their own funds to the experiment bank account. Participants who could not use this automated system were able to transfer the required amount manually to the experiment bank account. The experiment bank account was monitored closely, and when a deposit payment was received, we confirmed this to the participant through the intervention app. If no payment was received, participants were automatically reminded via push messages, SMS text messages, telephone calls, and email reminders. Participants were excluded if deposit payments were not confirmed 12 hours before the start of the intervention. After confirming the received deposit payment, we instructed the participants to wait until the intervention started the next Monday morning.

#### Condition 5: Deposit and Loss Frame Condition

Participants in this condition followed the same overall procedure as the participants in the deposit and gain frame condition did. However, to create the loss-framed feedback, they were informed that there was a full pot of €10 at the start of the intervention and that for every goal failure, €0.50 would be deducted from the pot. If they were successful, nothing would be deducted from the pot. The final pot amount was the amount of their deposit that we promised to return after the intervention.

### Debriefing

After the participants completed the 20-day intervention, they received a summary of their performance in the challenge. In the 4 experimental conditions, the participants were additionally informed about their incentive earnings and told that they would receive this money (back) into their bank account as soon as possible. Thereafter, the participants were sent a link to the survey platform LimeSurvey that opened within the Benefit Move app. Here, they filled in the final survey (for more details, see the Final Survey section) and returned to the intervention app after completion. Participants were then debriefed about their condition; the other conditions and the deceptive element around their deposit were revealed. All payments to the participants were made within 2 weeks after the experiment ended.

### Statistical Analysis

The primary outcome (continuous) was the effectiveness. This was measured through the mobile registration of step count data and defined as the number of days (0-20) the goal was achieved. The secondary outcome (binary) was the uptake of the intervention and defined as explicitly agreeing to participate in the challenge and paying the deposit (if required).

We report results on the effectiveness based on a restricted sample that only included participants who retrieved steps on at least one intervention day and who received a tailored step goal. We excluded participants who received a default goal, because in hindsight, these participants were confronted with a goal that was unachievable (Multimedia Appendix 4 provides an overview of analyses where these participants were included). Furthermore, we report the main analyses for effectiveness based on models that include baseline step counts as a covariate. The pattern of the results was similar, but the models gained accuracy by including the covariate. Data analysis was performed using SPSS statistics for Mac (version 28; IBM Corp). We dealt with missing cases by using pairwise exclusion and used the standard *P*<.05 criterion for determining statistical significance. For ANOVA and ANCOVA, we considered an effect size small when *ηp^2^*>0.01, medium when >0.06, and large when >0.14 (Cohen [55]). For chi-square, we considered an effect size small when Cramer *V*>0.1, medium when >0.3, and large when >0.5.

### Hypothesis Testing

#### Hypothesis 1: Effectiveness of Incentive Conditions Compared With the Control Condition

First, we performed an ANCOVA with baseline steps as a covariate in which we compiled incentive conditions to compare all incentive conditions combined (mean of conditions 2-5) to the control condition (ie, condition 1). Second, we performed an ANCOVA with baseline steps as a covariate and effectiveness as the dependent variable to separately compare incentive conditions (ie, conditions 2-5) to the no-incentive control condition (ie, condition 1). The ANCOVA was performed with factor “condition” with 5 levels (conditions 1-5). We compared each incentive group separately to the control condition with four planned contrasts: 1=control versus deposit and gain, 2=control versus deposit and loss, 3=control versus reward and gain, and 4=control versus reward and loss.

#### Hypothesis 2: Uptake of the Intervention

We performed a chi-square test of independence to investigate whether the uptake was lower for deposit contracts (ie, conditions 4 and 5) compared with regular rewards (ie, conditions 2 and 3).

#### Hypothesis 3 to 5: The Effect of Incentive Direction and Feedback Framing on Effectiveness

We performed a 2-way ANCOVA with baseline steps as a covariate. Effectiveness was the dependent variable, and the model contained 2 factors: incentive direction (deposit or reward) and feedback frame (loss or gain). In the model, we specified both the main effects of the factors (H2 and H3) and their interactions (H4).

## Results

### Descriptives

In total, we analyzed the data on the uptake of participants (N=126) with a mean age of 22.7 (SD 2.84) years of which 68.2% (86/126) identified as female. Most participants had the Dutch nationality (69/126, 54.8%), approximately half (60/126, 47.6%) were students, most reported to have an income similar to their peers (71/126, 56.3%), and most considered themselves to have an appropriate body weight (89/126, 70.6%). After their condition was explained to them, 11 participants explicitly refused the challenge, 7 participants did not pay their deposit in time, and 12 participants did not retrieve steps on any day of the intervention. Therefore, the data from 96 participants were available for the analysis of effectiveness, and the data from 65 participants remained after exclusion of nontailored goals (see the Methods section for rationale). Table 1 provides more details on the characteristics of the full sample that was analyzed for uptake and the subsample that was analyzed for effectiveness.

**Table 1:** Sample characteristics of the full sample and the subsample that was analyzed for effectiveness.

Variable			Full sample (N=126)		Subsample effectiveness (N=65)
Age (years), mean (SD)			22.7 (2.84)		22.2 (2.53)
**Sex, n (%)**					
	Male	40 (31.7)		13 (20)	
	Female	86 (68.3)		52 (80)	
**Nationality, n (%)**					
	Dutch	69 (54.8)		40 (61.5)	
	German	20 (15.9)		10 (15.4)	
	Other	37 (29.4)		15 (23.1)	
**Work** **, n (%)**					
	Student without a job	54 (42.8)		33 (50.8)	
	Student with a job	6 (4.8)		1 (1.5)	
	Working part time	14 (11.1)		6 (9.2)	
	Working full time	45 (35.7)		21 (32.3)	
	Do not want to answer	7 (5.6)		4 (6.2)	
**Income, n (%)**					
	Less than my peers	15 (11.9)		9 (13.8)	
	Same as my peers	71 (56.3)		39 (60)	
	More than my peers	20 (15.9)		9 (13.8)	
	Do not want to answer	20 (15.9)		8 (12.3)	
**Weight (kg)** **, n (%)**					
	Underweight	3 (2.4)		1 (1.5)	
	A bit underweight	7 (5.6)		4 (6.2)	
	Appropriate weight	89 (70.6)		48 (73.8)	
	A bit overweight	19 (15.1)		9 (13.8)	
	Overweight	7 (5.6)		2 (3.1)	
	Do not want to answer	1 (0.8)		1 (1.5)	

### Hypothesis Testing

#### Hypothesis 1: Effectiveness of Incentive Conditions Compared With Control Condition

First, a 1-way ANCOVA with baseline steps as a covariate showed that, overall, incentive conditions (mean 13.10, SD 6.33 days goal achieved) had higher effectiveness than the control condition (mean 8.00, SD 5.65 days goal achieved; *F*_1,62_=10.72; *P*=.002; *ηp^2^*=0.147). Furthermore, to test specific contrasts, a second 1-way ANCOVA with baseline steps as a covariate showed that the factor condition was related to the effectiveness of the intervention (*F*_4,59_=5.48; *P*<.001; *ηp^2^*=0.271). Participants in the control condition achieved their step goal on a mean of 8.00 (SD 5.65) days. Planned contrasts indicated that this was significantly less than that in the participants in reward and gain condition (mean 13.30, SD 5.49 days goal achieved; *P*=.003; SE 1.86). Furthermore, this was also significantly less than that of participants in the deposit and gain condition (mean 17.40, SD 6.17; *P*<.001; SE 2.25). We did not find a significant difference between the control condition and the reward and loss condition (mean 10.00, SD 7.01 days goal achieved; *P*=.23; SE 2.19). No significant difference was found between the control condition and the deposit and loss condition (mean 11.29, SD 5.16 days goal achieved; *P*=.19; SE 2.53). Owing to indications that normality of the dependent variable was violated, we performed a Kruskal-Wallis test to check the robustness of these findings. We only found a significant contrast between the control condition and the deposit and gain condition (*P*=.001, adjusted with Bonferroni correction). There was no evidence of a significant difference for the other contrasts.

#### Hypothesis 2: Uptake of the Intervention

Uptake of the intervention was defined as explicitly agreeing to participate in the challenge and paying the deposit (if required). A chi-square test of independence showed that requiring a deposit decreased the uptake of the intervention (N=97; *χ^2^*_1_=23.5; *P*<.001; Cramer *V*=0.492). In the reward conditions, 100% (50/50) of the participants accepted the intervention compared with 62% (29/47) in the deposit conditions (Table 2 provides a descriptive overview of the results). We explored whether those with uptake differed from those with no uptake but were underpowered for these analyses and accordingly found no differences in demographic data (sex, income, weight status, and age) or other baseline characteristics (goal type, self-efficacy, risk proneness, self-control, autonomous motivation, extrinsic motivation, and historic step count).

**Table 2:** Descriptive overview of the results.

Variable		Condition						Total (N=126)
		Control (n=29)	Reward and gain frame (n=32)	Reward and loss frame (n=18)	Deposit and gain frame (n=23)	Deposit and loss frame (n=24)		
Uptake, n (%)		29 (100)	32 (100)	18 (100)	15 (65)	14 (58)	108 (86)	
Explicit refusal, n (%)		0 (0)	0 (0)	0 (0)	4 (17)	7 (29)	11 (9)	
Deposit not paid, n (%)		N/A^a^	N/A	N/A	4 (17)	3 (12)	7 (6)	
Steps never retrieved, n (%)		2 (7)	4 (12)	3 (17)	0 (0)	3 (12)	12 (10)	
**Goal type, n (%)**								
	Tailored goals	18 (62)	21 (66)	11 (61)	17 (74)	14 (58)	81 (68)	
	Default goals 10,000	11 (38)	11 (34)	7 (39)	6 (26)	10 (42)	45 (36)	
Assigned step goal, mean (SD)		6189 (3604)	6384 (3700)	6992 (3111)	5960 (3544)	7714 (3724)	6602 (3574)	

^a^N/A: not applicable.

#### Hypothesis 3 to 5: Effect of Incentive Direction and Feedback Framing on Effectiveness

A 2-way ANCOVA with baseline steps as a covariate showed no main effect of incentive direction (*F*_1,43_=1.98; *P*=.17; *ηp^2^*=0.044), indicating that deposits (mean 14.88, SD 6.40 days goal achieved) were not more effective than rewards (mean 12.13, SD 6.17 days goal achieved). We did find a main effect of feedback framing (*F*_1,43_=7.91; *P*=.007; *ηp^2^*=0.155), indicating that loss frames (mean 10.50, SD 6.22 days goal achieved) were significantly less effective than gain frames (mean 14.67, SD 5.95 days goal achieved). Finally, the interaction effect of incentive direction×feedback framing was not significant (*F*_1,43_=1.16; *P*=.29; *ηp^2^*=0.026), indicating that feedback framing did not have a different effect on deposit conditions compared with reward conditions. Table 3 provides a descriptive overview of the results for each arm of the experiment.

Furthermore, to test the robustness of these findings, we additionally performed a Kruskal-Wallis test. For the main effects, we performed 2 separate tests, one for each factor from the 2-way ANOVA. However, the interaction effect could not be tested with this alternative method. Consistent with the results of the 2-way ANCOVA, we found that incentive direction was not significantly related to effectiveness (*P*=.06), but feedback framing was significantly related to effectiveness (*P*=.03). Additional checks to test the sensitivity of the main findings are reported in Multimedia Appendix 5.

**Table 3:** Descriptive overview of results for participants with tailored goals.

Variable	Condition, mean (SD)						Total (N=65), mean (SD)
	Control (n=17)	Reward and gain frame (n=20)	Reward and loss frame (n=11)	Deposit and gain frame (n=10)	Deposit and loss frame (n=7)		
Baseline step count	3406 (1982)	3868 (2673)	4232 (2056)	4036 (3187)	3472 (1537)	3792 (2347)	
Assigned step goal	4087 (2378)	4642 (3207)	5078 (2467)	4843 (3825)	4166 (1844)	4550 (2816)	
Intervention step count	3130 (2466)	5071 (2783)	4763 (2105)	6395 (4526)	3993 (2464)	4599 (3025)	
Days goal achieved	8.00 (5.65)	13.30 (5.49)	10.00 (7.01)	17.40 (6.17)	11.29 (5.16)	11.77 (6.52)	

### Effect of the Manipulations on Experienced Feelings of Loss and Goal Commitment

To check the effect of our manipulations, we analyzed the effects of incentive direction and feedback framing on feelings of loss and goal commitment. We performed 2 separate 2-way ANOVAs (one for feeling of loss and one for goal commitment) with factor incentive direction (deposit or reward) and factor feedback frame (loss or gain). The model included both main effects and their interactions. The first ANOVA, with feeling of loss as the dependent variable, showed a significant effect of incentive direction (*F*_1,41_=19.66; *P*<.001; *ηp^2^*=0.324). Deposit contracts (mean 7.19, SD 2.23) resulted in stronger feelings of loss compared with rewards (mean 4.21, SD 2.19). However, feedback framing did not influence the feeling of loss, and we did not find a significant interaction. The second ANOVA, with goal commitment as the dependent variable, showed a significant effect of feedback framing (*F*_1,41_=4.95; *P*=.03; *ηp^2^*=0.108). Loss-framed incentives (mean 5.24, SD 3.11) resulted in weaker goal commitment compared with gain-framed incentives (mean 7.14, SD 2.37). However, incentive direction did not influence goal commitment, and we did not find any interaction.

## Discussion

### Principal Findings

This study found that financial incentives increase intervention effects compared with an active no-incentive control condition. Furthermore, as expected, the results showed that self-funded deposit contracts for physical activity have a lower uptake than regular reward incentives. However, in contrast to our hypothesis, we did not find deposit contracts to be more effective than reward incentives, but they were also not less effective and have important benefits for large-scale implementation. An important unexpected finding was that loss framing decreased the effectiveness of the intervention compared with gain framing. This finding is in contrast to the existing literature and seems to provide the first preliminary evidence that for improving physical activity with financial incentives in a healthy population, loss framing is less effective than gain framing.

First, the finding that financial incentive conditions were more effective than an active no-incentive control condition is in line with the results from meta-analyses [14-16]. Compared with participants in the control condition, participants who received a financial incentive were shown to reach about 5 more daily step goals (and took about 2000 steps more per day) during the 20-day intervention. This is a large and clinically relevant effect with a mortality-reducing potential [5,6]. We explain this finding through the idea that financial incentives capitalize on the present bias and introduce an immediate monetary incentive for being physically active.

Second, we found that the uptake of deposit contracts was lower than that of regular rewards. This finding is in line with the work by Halpern et al [19] on deposit contracts for smoking cessation. A common sense explanation for this finding is that people are more open to an intervention where they stand to gain something (ie, a reward) than where they stand to lose something (ie, their own money). The same aversion to losses that is thought to increase effectiveness might deter people from entering into a deposit contract. In fact, this tension between effectiveness and uptake has been recognized before [56]. Furthermore, although we simplified all steps in the payment process, it could be that the logistical barrier of having to provide a monetary deposit deterred some individuals, regardless of whether they dismissed the concept of deposit contracts per se. Finally, it is important to understand which people are most likely to accept and reject a deposit contract intervention. For example, it has previously been suggested that individuals who recognize their challenges while resisting temptation (ie, sophisticates) might be open to using deposit contracts [56]. Future research should use a self-funded deposit contract and investigate the moderators of uptake to shed light on which subgroups are best reached.

Third, in contrast to our hypothesis, deposit contracts were not more effective than regular reward incentives. We expected, in line with others, that deposit contracts would invoke loss aversion and therefore would be more effective than regular rewards. Our analyses indeed showed that deposit contracts resulted in stronger feelings of loss than rewards did, but this did not result in higher effectiveness. Our results are in contrast to those reported for smoking cessation by Halpern et al [19]. Possibly, for physical activity, deposit contracts are not more effective than rewards. Another explanation might be that participants perceived the stakes in our study as low and therefore were not averse to potentially losing their deposits. This would be in line with the work by Mukherjee et al [57] who found that for high stakes, participants rated losses more impactful than gains (ie, loss aversion), but for low stakes, this tendency reversed, and gains were rated as more impactful than losses. It is possible that subjective judgments by our participants rated the incentive as low stakes and therefore deposit contracts were not more effective than rewards. Future work should investigate deposit contracts and rewards of varying sizes to determine the potential tipping points at which deposit contracts are superior to rewards and when this is reversed. In addition, it is possible that deposit contracts are superior to rewards (the descriptive means were in the expected direction), but we did not have enough statistical power to detect a significant difference. More fully powered studies that investigate self-funded deposit contracts for physical activity are needed to draw firmer conclusions on this point. Existing studies in the domain of physical activity either operationalized deposit contracts differently using loss framing [20,24] or were also not powered [21,28-30] to provide a clear answer to this question.

Finally, unexpectedly, we found that loss framing decreased the effectiveness of the intervention compared with gain framing. In line with the study by Patel et al [20], we expected that framing an incentive as a loss would activate loss aversion and therefore increase effectiveness compared with gain framing an incentive. However, our analyses showed that loss framing did not increase feelings of loss compared with gain framing. Thus, it appears that our attempt at shifting participants’ reference point was unsuccessful. We did find that loss framing decreased feelings of goal commitment, which might explain why the effectiveness of loss frames was lower than that of gain frames. Our results contradict the findings of Patel et al [20] who showed that loss-framed incentives were more effective than gain-framed incentives. However, Patel et al [20] studied university employees who are obese, with a BMI >27, whereas our sample consisted of healthy university students. Possibly, a difference in regulatory fit related to differences in the study sample might explain this discrepancy. Regulatory fit is when the persuasiveness of a health message is increased when its frame is congruent with the regulatory orientation of the individual [58]. Regulatory focus theory discerns 2 modes of regulatory orientation: promotion focus and prevention focus. Although people with a promotion focus aim for desired end states, people with a prevention focus aim for avoiding undesired end states [58]. Perhaps, adults who are obese are more focused on avoiding obesity-related health problems, and therefore have a stronger prevention focus when increasing physical activity. This could lead them to respond better to a loss-framed incentive (in which losing money is prevented) because of a greater experienced regulatory fit. By contrast, perhaps healthy students have a stronger promotion focus (on becoming more fit rather than avoiding health problems) and therefore respond better to a gain-framed incentive. Whether the regulatory fit effect also applies to incentive framing (and not only to framing of persuasive health messages) is an interesting avenue for future research. Future research should measure regulatory orientation and investigate the possible interactions with different incentive frames.

### Strengths and Limitations

An important strength of this study is that we used a self-funded deposit contract that required participants to make a monetary deposit before the intervention started. This allowed us to compare the effects of self-funded deposit contracts with those of loss frames. Another strength is that we used objective registrations of step counts and did not rely on self-reported estimations of physical activity. Finally, the app automatically provided participants with tailored goals based on their historical step counts, thus creating a personalized intervention experience. However, requiring a deposit beforehand also resulted in a lower uptake of the deposit contract conditions. As a result, the deposit requirement may have filtered out people who lacked motivation, thus leading to an overestimation of effectiveness in the deposit contract conditions. Consequently, caution is warranted when interpreting the effectiveness of the deposit contract conditions. Another limitation of our study is that high dropout before onboarding, unbalanced allocation, lack of uptake in the deposit contract conditions, and the exclusion of nontailored goals decreased the statistical power of our analyses. Limited statistical power might have especially affected the findings for specific analyses on effectiveness such as when we compare deposit contracts with regular rewards or loss frames with gain frames. Therefore, the results of this study should be interpreted with caution, and future work should be done to confirm these findings. Furthermore, before onboarding, participants read the informed consent form, which mentioned that the study possibly required them to deposit €10 of their own money. Mentioning this possibility was important for informed consent but may have deterred some participants from participating before they onboarded in the app. It is possible that this biased our analysis of uptake and that the actual uptake of deposit contracts is lower than our analyses suggest. In addition, although we propose that objective measures of physical activity are superior to subjective self-reports, an important criticism of pedometer-based intervention research is that it is impossible to differentiate an increase in step count from an increase in pedometer wear time [59]. In our case, participants in the gain-framed conditions reported having carried their smartphone more often than they normally do (Multimedia Appendix 5), and this might partly explain why gain-framed conditions were more effective than loss-framed conditions. Furthermore, a relatively high proportion of the participants (45/126, 35.7%) did not have historical step data available on their smartphones. These people were assigned a default goal (10,000 steps per day) that was unachievable in hindsight. Although 10,000 steps per day is often used as a goal in commercial physical activity trackers and apps, this already exceeds the guidelines for sufficient physical activity, which translates to approximately 7000 to 8000 steps per day [60]. Future research with a similar goal-setting module should assign more achievable default goals when the goals cannot be tailored. In our sample, the mean baseline step count of participants with historical data was approximately 3800 steps per day. On the basis of a meta-analysis of financial incentive intervention effects, we suggest that step goals should not exceed baseline levels by >20% to 30% [14]. In addition, the intervention was launched in March 2020, and during this period, the first COVID-19 lockdown measures in the Netherlands were implemented. Although this probably impacted all conditions equally, a large part (51/65, 78%) of the sample reported having been less physically active than they normally were because of the situation around COVID-19. As a result, it is possible that the estimates of baseline activity were lower than normal; therefore, the intervention led to stronger improvements than would be found under normal circumstances. Furthermore, our sample consisted of predominantly healthy, young, female students at universities. Although we purposefully recruited a homogenous sample to increase internal validity, the external validity of our findings is therefore restricted. Older or more chronically ill populations might respond differently to this type of intervention. Finally, we only investigated short-term effects during a 20-day intervention period. Therefore, we are unable to answer questions about the long-term effectiveness of the different incentive directions and incentive frames that we tested. Future work with longer intervention durations should be done to study how rates of goal achievement (and step counts) vary over time during and after the intervention.

### Implications

An important theoretical contribution of this study is that we did not replicate the finding that loss-framed financial incentives are more effective than gain-framed financial incentives for increasing physical activity [20]. By contrast, our results show that gain-framed incentives are more effective. Although we are unable to ascertain what has produced this effect, by itself it provides evidence that (perceptions of) losses are not always more impactful than (perceptions of) gains. Rather, it supports the argument made by Gal and Rucker [61] that loss aversion is a context-dependent tendency with boundary conditions, instead of a ubiquitous phenomenon. This finding also has implications for those who want to implement loss-framed financial incentives in practice. Because our results show that loss frames might hurt incentive effectiveness, we warn against implementing them in practice without further research on their boundary conditions. Finally, we were unable to show that deposit contracts were more effective than rewards, but they were also not less effective. Considering that deposit contracts are (partially) self-funded makes them attractive for large-scale implementation. However, before deposit contracts can be implemented on a large scale, it is important to further understand which subgroups are not reached by them. Although to the best of our knowledge the relationship between income and uptake of deposit contracts has not yet been studied, one can imagine that people with lower incomes might reject a deposit contract because they are less able to deposit a sum of their own money. This could cause vulnerable key subgroups (eg, people with lower socioeconomic status or cardiovascular disease) not to be reached by a deposit contract intervention. Possibly, this issue could be overcome by offering income-dependent deposit sizes or allowing participants to freely choose an amount that is motivating but that does not cause financial harm when lost [26].

### Conclusions

Although this study was underpowered and the results have to be interpreted with caution, we have shown that deposit contracts have lower uptake than rewards but appear to have (at least) comparable effects on physical activity. Loss framing an incentive might undermine effectiveness, and we therefore urge for more research before implementing them in practice. Deposit contracts might be a promising tool for behavior change; however, more research is needed on who is willing to use them and for whom they are most effective.

## Appendix

Multimedia Appendix 1 Flowchart.

Multimedia Appendix 2 Baseline survey.

Multimedia Appendix 3 Final survey.

Multimedia Appendix 4 Main analysis total sample.

Multimedia Appendix 5 Sensitivity checks.

Multimedia Appendix 6 CONSORT-eHEALTH checklist (V 1.6.1).

## Abbreviations

ANCOVA: analysis of covariance
