# Supplementary figures and images for: Investigating Rewards and Deposit Contract Financial Incentives for Physical Activity Behavior Change Using a Smartphone App: Randomized Controlled Trial
Source: J Med Internet Res. 2022 Oct 6;24(10):e38339. doi: 10.2196/38339 (PMC11042509; doi:10.2196/38339)

**Appendix A: Flowchart**


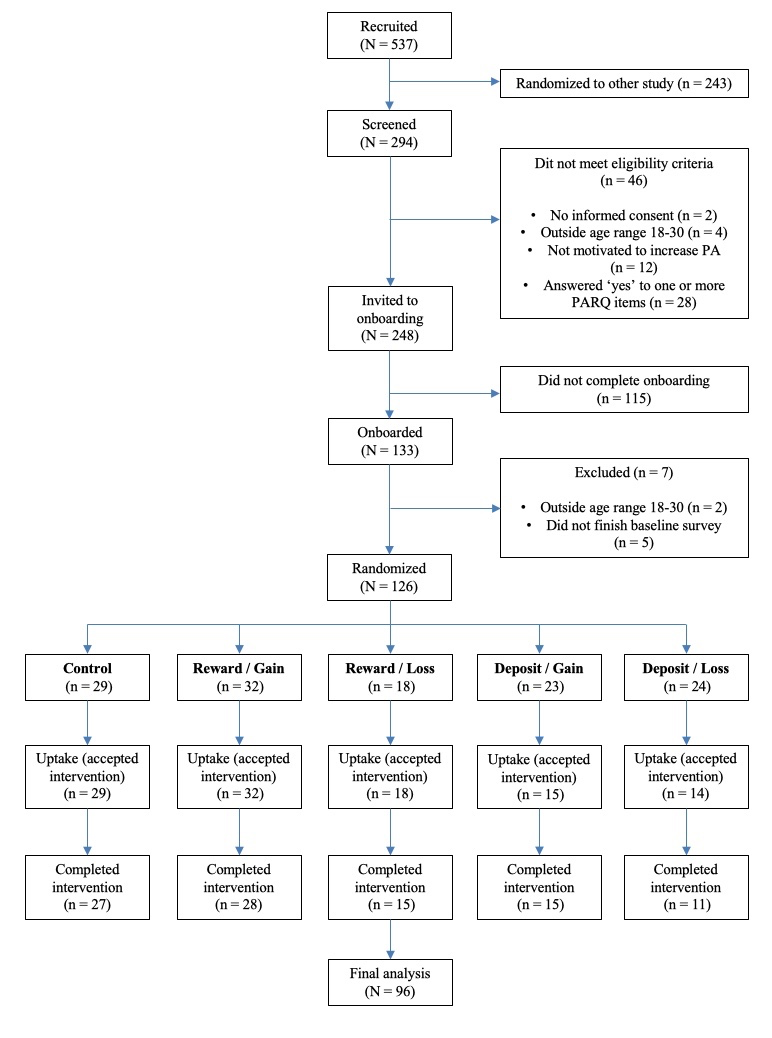

Supplement: Multimedia Appendix 1 [file jmir_v24i10e38339_app1.docx]
