# Supplementary material for: Investigating Rewards and Deposit Contract Financial Incentives for Physical Activity Behavior Change Using a Smartphone App: Randomized Controlled Trial
Source: J Med Internet Res. 2022 Oct 6;24(10):e38339. doi: 10.2196/38339 (PMC11042509; doi:10.2196/38339)
Supplement: Multimedia Appendix 2 [file jmir_v24i10e38339_app2.docx]

**Appendix B: Baseline survey original items**

1. What gender do you identify with?

- Man
- Woman
- Other

2. What is your birth year?

3. What is your Nationality?

4. In which country do you currently live?

5. Do you currently study at a university or a university of applied sciences (HBO)?

- Yes
- No

6. Please select which of the following options is most applicable to you

- Working full-time
- Working part-time
- Student with a job
- Student without a job
- I don't want to answer this question

7. How much money do you have available?

- More than my fellow students
- About the same as my fellow students
- Less than my fellow students
- I don't want to answer this question

8. Which of the categories below do you self-identify with?

- Underweight
- A bit underweight
- At an appropriate weight
- A bit overweight
- Overweight
- I don't want to answer this question
