# Supplementary material for: Investigating Rewards and Deposit Contract Financial Incentives for Physical Activity Behavior Change Using a Smartphone App: Randomized Controlled Trial
Source: J Med Internet Res. 2022 Oct 6;24(10):e38339. doi: 10.2196/38339 (PMC11042509; doi:10.2196/38339)
Supplement: Multimedia Appendix 3 [file jmir_v24i10e38339_app3.docx]

**Appendix C: Final survey original items**

1. How frequently have you checked your step- count in the previous 20 days?

- 1 = Not at all
- 2 = Several days
- 3 = More than half the days
- 4 = Nearly every day
- 5 = Many times a day

2. How frequently have you checked how close you were to achieving your step goal in the past 20 days?

- 1 = Not at all
- 2 = Several days
- 3 = More than half the days
- 4 = Nearly every day
- 5 = Many times a day

3. Please indicate to what extent you agree with the three statements below. Please tick a box on the scale, where the value 1 means: ‘totally disagree’ and the value 10 means: ‘totally agree’.

- I felt that I was losing money if I did not increase my step count
- I felt strongly committed to the goal of increasing my step count
- Because I participated in this study I chose to carry my smartphone more with me than I would normally do

4. Did you cheat the system in any way (do not worry, you will receive your incentive as promised, independent of your answer to this question) ?

- Yes
- No

5. Did you know other people who were also participating in this experiment?

- Yes
- No

6. Did you discuss the experiment with them?

- Yes
- No

7. Did you know about what they were required to do for the study?

- Yes
- No

8. Could you please write down what the other participant(s) were required to do in the experiment?

9. Did you experience flu-like symptoms like fever, coughing and/or trouble breathing during the intervention period of this study?

- Yes
- No

10. Did you engage in less physical activity due to these symptoms?

- Yes
- No

11. Did you engage in less physical activity than usual due to the current situation with regard to the Corona-virus?

- Yes
- No

12. Over the last three weeks, how often have you been bothered by the following problems?

(1 = Not at all, 2 = Several days, 3 = More than half the days, 4 = Nearly every day)

- Feeling nervous, anxious or on edge
- Not being able to stop or control worrying
- Worrying too much about different things
- Trouble relaxing
- Being so restless that it is hard to sit still
- Becoming easily annoyed or irritable
- Feeling afraid as if something awful might happen

13. If you checked off any problems, how difficult have these problems made it for you to do your work, take care of things at home or get along with other people?

- This does not apply
- 1 = Not difficult at all
- 2 = Somewhat difficult
- 3 = Very difficult
- 4 = Extremely difficult
