# Supplementary material for: Investigating Rewards and Deposit Contract Financial Incentives for Physical Activity Behavior Change Using a Smartphone App: Randomized Controlled Trial
Source: J Med Internet Res. 2022 Oct 6;24(10):e38339. doi: 10.2196/38339 (PMC11042509; doi:10.2196/38339)
Supplement: Multimedia Appendix 4 [file jmir_v24i10e38339_app4.docx]

**Appendix D: Main analysis on effectiveness for total sample**

*In this appendix we report the main analysis on effectiveness for the total sample (N = 96). In the running text we report analyses on a restricted sample that only included participants who received a tailored step goal (N = 65). We chose to exclude the participants that received a default 10.000 step goal. In hindsight, these participants were confronted with a goal that was unachievable because it was set at more than 250% of their baseline step count. In contrast, the average baseline step count for participants who did receive a tailored goal was 3792 steps per day and tailored goals were set at 120% of baseline to remain achievable. To be complete we report the main analyses for the full sample (including those who received default goals) here.*

***Hypothesis 1: Effectiveness of incentive conditions compared to control condition***

A one-way ANCOVA with baseline steps as covariate showed that the factor condition was not significantly related to the effectiveness of the intervention, *F*(4, 90) = 1.90, *p* = .117, *ηp2* = .078. Planned contrasts were not further investigated because the multivariate test was not significant.

***Hypothesis 3-4-5: The effect of incentive direction and feedback framing on effectiveness***

A two-way ANCOVA with baseline steps as covariate showed no main effect of incentive direction, *F*(1, 64) = 1.861, *p* = .177, *ηp2* = .028 indicating that deposits (*M* = 11.46, *SD* = 7.86) were not more effective than rewards (*M* = 9.49, SD = 7.01). There was no main effect of feedback framing, *F*(1, 64) = 1.571, *p* = .215, *ηp2* = .024 indicating that loss frames (*M* = 8.81, *SD* = 6.68) were not significantly more effective than gain frames (*M* = 11.09, *SD* = 7.67). Finally, the interaction effect of incentive direction * feedback framing was not significant, *F*(1, 64) = .135, *p* = .714, *ηp2* = .002 indicating that feedback framing did not have a different effect in deposit conditions compared to in reward conditions. See *Table 1* for a descriptive overview of the results per arm of the experiment.

| **Table 1.** *Descriptive overview of results for full sample (N = 96)*   \| Variable \| Condition \| \| \| \| \|  \| \| --- \| --- \| --- \| --- \| --- \| --- \| --- \| \|  \| **Control** \| **Reward / Gain frame** \| **Reward / Loss frame** \| **Deposit / Gain frame** \| **Deposit / Loss frame** \| **Total** \| \| **N** \| 27 \| 28 \| 15 \| 15 \| 11 \| 96 \| \| **Baseline step count** \| 2144 (2287) \| 2763 (2863) \| 3103 (2603) \| 2690 (3226) \| 2209 (2118) \| 2567 (2625) \| \| **Assigned step goal** \| 6277 (3457) \| 6173 (3649) \| 6390 (3070) \| 6562 (3967) \| 6287 (3272) \| 6310 (3450) \| \| **Intervention step count** \| 3770 (3290) \| 4539 (3048) \| 4494 (2075) \| 5343 (4151) \| 5143 (2979) \| 4511 (3166) \| \| **Days goal achieved** \| 6.78 (6.45) \| 10.46 (6.83) \| 7.67 (7.21) \| 12.27 (9.18) \| 10.36 (5.84) \| 9.3 (7.2) \|   Note: data are means (SD) |
| --- | --- | --- | --- | --- | --- | --- | --- | --- | --- | --- | --- | --- | --- | --- | --- | --- | --- | --- | --- | --- | --- | --- | --- | --- | --- | --- | --- | --- | --- | --- | --- | --- | --- | --- | --- | --- | --- | --- | --- | --- | --- | --- | --- | --- | --- | --- | --- | --- | --- |
