# Supplementary material for: Investigating Rewards and Deposit Contract Financial Incentives for Physical Activity Behavior Change Using a Smartphone App: Randomized Controlled Trial
Source: J Med Internet Res. 2022 Oct 6;24(10):e38339. doi: 10.2196/38339 (PMC11042509; doi:10.2196/38339)
Supplement: Multimedia Appendix 5 [file jmir_v24i10e38339_app5.docx]

**Appendix E: Sensitivity checks**

We performed several additional checks to test the sensitivity of the main findings to possible cheating, anxiety symptoms (related to the COVID-19 lockdown), experienced COVID-19 symptoms, whether participants were less active due to COVID-19, contamination between conditions and whether people carried their smartphone more often. Six individuals reported to have cheated the intervention and indeed showed significantly higher goal achievement. However, after removing these individuals from the sample, the pattern of results was not affected. Anxiety symptoms measured with GAD-7 were not correlated to goal achievement and thus considered not problematic. 8 participants (12.3%) reported having been less physically active due to flu-like symptoms and 51 participants (78.5%) reported to have been less physically active due to the situation around COVID-19. With regard to contamination between conditions, 24 (38.7%) participants knew others who also participated in the experiment. Of these participants, 11 (16.9%) also knew what other participants were required to do for the study. However, we did not find a relationship between knowing others and effectiveness of the intervention. Finally, participants scored relatively high on a 10-point Likert scale when asked whether they carried their smartphone more often due to the experiment (*M* = 6.6, *SD* = 3.20), and there was a significant correlation between carrying the smartphone more often and goal achievement (*r* = .473, *p* < .001). Furthermore, a two-way ANOVA with factor incentive direction, factor feedback framing and their interaction showed a significant effect of feedback framing on carrying the smartphone more often (*F*(1, 40) = 5.23, *p* = .028, *ηp2* = .116). Participants who received a loss-framed incentive carried their smartphone less often (*M* = 5.44, *SD* = 3.39) than participants who received a gain-framed incentive (*M* = 7.82, *SD* = 2.57). There was no effect of incentive direction on carrying the smartphone more often nor did we find an interaction.
