# Supplementary material for: Investigating Rewards and Deposit Contract Financial Incentives for Physical Activity Behavior Change Using a Smartphone App: Randomized Controlled Trial
Source: J Med Internet Res. 2022 Oct 6;24(10):e38339. doi: 10.2196/38339 (PMC11042509; doi:10.2196/38339)
Supplement: Multimedia Appendix 6 [file jmir_v24i10e38339_app6.pdf]

CONSORT-EHEALTH (V 1.6.1) - Submission/Publication Form

The CONSORT-EHEALTH checklist is intended for authors of randomized trials evaluating web-based and Internet-based applications/interventions, including mobile interventions, electronic games (incl multiplayer games), social media, certain telehealth applications, and other interactive and/or networked electronic applications. Some of the items (e.g. all subitems under item 5 - description of the intervention) may also be applicable for other study designs.

The goal of the CONSORT EHEALTH checklist and guideline is to be
a) a guide for reporting for authors of RCTs,
b) to form a basis for appraisal of an ehealth trial (in terms of validity)

CONSORT-EHEALTH items/subitems are MANDATORY reporting items for studies published in the Journal of Medical Internet Research and other journals / scientific societies endorsing the checklist.

Items numbered 1., 2., 3., 4a., 4b etc are original CONSORT or CONSORT-NPT (non-pharmacologic treatment) items.
Items with Roman numerals (i., ii, iii, iv etc.) are CONSORT-EHEALTH extensions/clarifications.

As the CONSORT-EHEALTH checklist is still considered in a formative stage, we would ask that you also RATE ON A SCALE OF 1-5 how important/useful you feel each item is FOR THE PURPOSE OF THE CHECKLIST and reporting guideline (optional).

Mandatory reporting items are marked with a red *.
In the textboxes, either copy & paste the relevant sections from your manuscript into this form - please include any quotes from your manuscript in QUOTATION MARKS,
or answer directly by providing additional information not in the manuscript, or elaborating on why the item was not relevant for this study.

YOUR ANSWERS WILL BE PUBLISHED AS A SUPPLEMENTARY FILE TO YOUR PUBLICATION IN JMIR AND ARE CONSIDERED PART OF YOUR PUBLICATION (IF ACCEPTED).
Please fill in these questions diligently. Information will not be copyedited, so please use proper spelling and grammar, use correct capitalization, and avoid abbreviations.

DO NOT FORGET TO SAVE AS PDF _AND_ CLICK THE SUBMIT BUTTON SO YOUR ANSWERS ARE IN OUR DATABASE !!!

Citation Suggestion (if you append the pdf as Appendix we suggest to cite this paper in the

caption):
Eysenbach G, CONSORT-EHEALTH Group
CONSORT-EHEALTH: Improving and Standardizing Evaluation Reports of Web-based and Mobile Health Interventions
J Med Internet Res 2011;13(4):e126
URL: http://www.jmir.org/2011/4/e126/
doi: 10.2196/jmir.1923
PMID: 22209829

davidbuisonje@gmail.com (niet gedeeld) Ander account Concept opgeslagen

*Vereist

Your name *

First Last

David de Buisonjé

Primary Affiliation (short), City, Country *

University of Toronto, Toronto, Canada

Leiden University, Leiden, The Netherlands

Your e-mail address *

abc@gmail.com

d.r.de.buisonje@fsw.leidenuniv.nl

Title of your manuscript *

Provide the (draft) title of your manuscript.

Less carrot more stick? Investigating rewards and deposit contract financial incentives for physical activity behavior change using a smartphone application: randomised controlled trial

Name of your App/Software/Intervention *

If there is a short and a long/alternate name, write the short name first and add the long name in brackets.

MobileCoach

Evaluated Version (if any)

e.g. "V1", "Release 2017-03-01", "Version 2.0.27913"

Jouw antwoord

Language(s) *

What language is the intervention/app in? If multiple languages are available, separate by comma (e.g. "English, French")

English

URL of your Intervention Website or App

e.g. a direct link to the mobile app on app in appstore (itunes, Google Play), or URL of the website. If the intervention is a DVD or hardware, you can also link to an Amazon page.

www.mobile-coach.eu

URL of an image/screenshot (optional)

Jouw antwoord

Accessibility *

Can an enduser access the intervention presently?

- ○ access is free and open
- ○ access only for special usergroups, not open
- ○ access is open to everyone, but requires payment/subscription/in-app purchases
- ◉ app/intervention no longer accessible
- ○ Anders:

Primary Medical Indication/Disease/Condition *

e.g. "Stress", "Diabetes", or define the target group in brackets after the condition, e.g. "Autism (Parents of children with)", "Alzheimers (Informal Caregivers of)"

Healthy population

Primary Outcomes measured in trial *

comma-separated list of primary outcomes reported in the trial

Effectiveness (continuous: days goal achieved

Secondary/other outcomes

Are there any other outcomes the intervention is expected to affect?

Uptake of the intervention (binary)

Recommended "Dose" *

What do the instructions for users say on how often the app should be used?

- (x) Approximately Daily
- ( ) Approximately Weekly
- ( ) Approximately Monthly
- ( ) Approximately Yearly
- ( ) "as needed"
- ( ) Anders:

Approx. Percentage of Users (starters) still using the app as recommended after 3 months *

- [x] unknown / not evaluated
- [ ] 0-10%
- [ ] 11-20%
- [ ] 21-30%
- [ ] 31-40%
- [ ] 41-50%
- [ ] 51-60%
- [ ] 61-70%
- [ ] 71%-80%
- [ ] 81-90%
- [ ] 91-100%
- [ ] Anders:

Overall, was the app/intervention effective? *

- (x) yes: all primary outcomes were significantly better in intervention group vs control
- ( ) partly: SOME primary outcomes were significantly better in intervention group vs control
- ( ) no statistically significant difference between control and intervention
- ( ) potentially harmful: control was significantly better than intervention in one or more outcomes
- ( ) inconclusive: more research is needed
- ( ) Anders:

Article Preparation Status/Stage *

At which stage in your article preparation are you currently (at the time you fill in this form)

- ( ) not submitted yet - in early draft status
- ( ) not submitted yet - in late draft status, just before submission
- (x) submitted to a journal but not reviewed yet
- ( ) submitted to a journal and after receiving initial reviewer comments
- ( ) submitted to a journal and accepted, but not published yet
- ( ) published
- ( ) Anders:

Journal *

If you already know where you will submit this paper (or if it is already submitted), please provide the journal name (if it is not JMIR, provide the journal name under "other")

- ○ not submitted yet / unclear where I will submit this
- ◉ Journal of Medical Internet Research (JMIR)
- ○ JMIR mHealth and UHealth
- ○ JMIR Serious Games
- ○ JMIR Mental Health
- ○ JMIR Public Health
- ○ JMIR Formative Research
- ○ Other JMIR sister journal
- ○ Anders:

Is this a full powered effectiveness trial or a pilot/feasibility trial? *

- ○ Pilot/feasibility
- ◉ Fully powered

Manuscript tracking number *

If this is a JMIR submission, please provide the manuscript tracking number under "other" (The ms tracking number can be found in the submission acknowledgement email, or when you login as author in JMIR. If the paper is already published in JMIR, then the ms tracking number is the four-digit number at the end of the DOI, to be found at the bottom of each published article in JMIR)

- ○ no ms number (yet) / not (yet) submitted to / published in JMIR
- ◉ Anders: ms#38339

TITLE AND ABSTRACT

1a) TITLE: Identification as a randomized trial in the title

1a) Does your paper address CONSORT item 1a? *

I.e does the title contain the phrase "Randomized Controlled Trial"? (if not, explain the reason under "other")

- ◉ yes
- ○ Anders:

1a-i) Identify the mode of delivery in the title

Identify the mode of delivery. Preferably use “web-based” and/or “mobile” and/or “electronic game” in the title. Avoid ambiguous terms like “online”, “virtual”, “interactive”. Use “Internet-based” only if Intervention includes non-web-based Internet components (e.g. email), use “computer-based” or “electronic” only if offline products are used. Use “virtual” only in the context of “virtual reality” (3-D worlds). Use “online” only in the context of “online support groups”. Complement or substitute product names with broader terms for the class of products (such as “mobile” or “smart phone” instead of “iphone”), especially if the application runs on different platforms.

	1	2	3	4	5	
subitem not at all important	○	○	○	○	◉	essential

Selectie wissen

Does your paper address subitem 1a-i? *

Copy and paste relevant sections from manuscript title (include quotes in quotation marks "like this" to indicate direct quotes from your manuscript), or elaborate on this item by providing additional information not in the ms, or briefly explain why the item is not applicable/relevant for your study

"using a smartphone application"

1a-ii) Non-web-based components or important co-interventions in title

Mention non-web-based components or important co-interventions in title, if any (e.g., “with telephone support”).

	1	2	3	4	5	
subitem not at all important	○	○	○	○	◉	essential

Selectie wissen

Does your paper address subitem 1a-ii?

Copy and paste relevant sections from manuscript title (include quotes in quotation marks "like this" to indicate direct quotes from your manuscript), or elaborate on this item by providing additional information not in the ms, or briefly explain why the item is not applicable/relevant for your study

"rewards and deposit contract financial incentives"

1a-iii) Primary condition or target group in the title

Mention primary condition or target group in the title, if any (e.g., “for children with Type I Diabetes”) Example: A Web-based and Mobile Intervention with Telephone Support for Children with Type I Diabetes: Randomized Controlled Trial

	1	2	3	4	5	
subitem not at all important	○	○	◉	○	○	essential

Selectie wissen

Does your paper address subitem 1a-iii? *

Copy and paste relevant sections from manuscript title (include quotes in quotation marks "like this" to indicate direct quotes from your manuscript), or elaborate on this item by providing additional information not in the ms, or briefly explain why the item is not applicable/relevant for your study

we targeted healthy students but did not include this in title due to word limit

1b) ABSTRACT: Structured summary of trial design, methods, results, and conclusions

NPT extension: Description of experimental treatment, comparator, care providers, centers, and blinding status.

1b-i) Key features/functionalities/components of the intervention and comparator in the METHODS section of the ABSTRACT

Mention key features/functionalities/components of the intervention and comparator in the abstract. If possible, also mention theories and principles used for designing the site. Keep in mind the needs of systematic reviewers and indexers by including important synonyms. (Note: Only report in the abstract what the main paper is reporting. If this information is missing from the main body of text, consider adding it)

	1	2	3	4	5	
subitem not at all important	○	○	○	○	◉	essential

Selectie wissen

Does your paper address subitem 1b-i? *

Copy and paste relevant sections from the manuscript abstract (include quotes in quotation marks "like this" to indicate direct quotes from your manuscript), or elaborate on this item by providing additional information not in the ms, or briefly explain why the item is not applicable/relevant for your study

"Healthy participants (N = 126) with an average age of 22.7 years participated in a 20-day physical activity intervention. They downloaded a smartphone application that provided them with a personalised physical activity goal and either required a €10 deposit upfront (which could be lost) or provided €10 as a reward, contingent on performance. Daily feedback on incentive earnings was provided and framed as either a loss or a gain. We employed a 2 (incentive type: deposit vs reward) x 2 (feedback frame: gain vs loss) between-subjects factorial design with a no incentive control condition. Our primary outcome was the number of days participants achieved their goal. Uptake of the intervention was a secondary outcome."

1b-ii) Level of human involvement in the METHODS section of the ABSTRACT

Clarify the level of human involvement in the abstract, e.g., use phrases like “fully automated” vs. “therapist/nurse/care provider/physician-assisted” (mention number and expertise of providers involved, if any). (Note: Only report in the abstract what the main paper is reporting. If this information is missing from the main body of text, consider adding it)

	1	2	3	4	5	
subitem not at all important	○	○	◉	○	○	essential

Selectie wissen

Does your paper address subitem 1b-ii?

Copy and paste relevant sections from the manuscript abstract (include quotes in quotation marks "like this" to indicate direct quotes from your manuscript), or elaborate on this item by providing additional information not in the ms, or briefly explain why the item is not applicable/relevant for your study

Because the intervention was almost fully automated we did not elaborate on this

1b-iii) Open vs. closed, web-based (self-assessment) vs. face-to-face assessments in the METHODS section of the ABSTRACT

Mention how participants were recruited (online vs. offline), e.g., from an open access website or from a clinic or a closed online user group (closed usergroup trial), and clarify if this was a purely web-based trial, or there were face-to-face components (as part of the intervention or for assessment). Clearly say if outcomes were self-assessed through questionnaires (as common in web-based trials). Note: In traditional offline trials, an open trial (open-label trial) is a type of clinical trial in which both the researchers and participants know which treatment is being administered. To avoid confusion, use “blinded” or “unblinded” to indicated the level of blinding instead of “open”, as “open” in web-based trials usually refers to “open access” (i.e. participants can self-enrol). (Note: Only report in the abstract what the main paper is reporting. If this information is missing from the main body of text, consider adding it)

	1	2	3	4	5	
subitem not at all important	◉	○	○	○	○	essential

Selectie wissen

Does your paper address subitem 1b-iii?

Copy and paste relevant sections from the manuscript abstract (include quotes in quotation marks "like this" to indicate direct quotes from your manuscript), or elaborate on this item by providing additional information not in the ms, or briefly explain why the item is not applicable/relevant for your study

no because it was not considered crucial information

1b-iv) RESULTS section in abstract must contain use data

Report number of participants enrolled/assessed in each group, the use/uptake of the intervention (e.g., attrition/adherence metrics, use over time, number of logins etc.), in addition to primary/secondary outcomes. (Note: Only report in the abstract what the main paper is reporting. If this information is missing from the main body of text, consider adding it)

	1	2	3	4	5	
subitem not at all important	○	○	○	○	◉	essential

Selectie wissen

Does your paper address subitem 1b-iv?

Copy and paste relevant sections from the manuscript abstract (include quotes in quotation marks "like this" to indicate direct quotes from your manuscript), or elaborate on this item by providing additional information not in the ms, or briefly explain why the item is not applicable/relevant for your study

" Overall, financial incentive conditions (M = 13.10 days) had higher effectiveness than the control condition (M = 8.00 days), p = .002, ηp2 = .147. Deposit contracts had lower uptake (61.7%) than rewards (100%), p = <.001, V = .492. Furthermore, two-way ANCOVA showed that deposit contracts (M = 14.88 days) were not significantly more effective than rewards (M = 12.13 days), p = .166. Unexpectedly, loss frames (M = 10.50 days) were significantly less effective than gain frames (M = 14.67 days), p = .007, ηp2 = .155. "

1b-v) CONCLUSIONS/DISCUSSION in abstract for negative trials

Conclusions/Discussions in abstract for negative trials: Discuss the primary outcome - if the trial is negative (primary outcome not changed), and the intervention was not used, discuss whether negative results are attributable to lack of uptake and discuss reasons. (Note: Only report in the abstract what the main paper is reporting. If this information is missing from the main body of text, consider adding it)

	1	2	3	4	5	
subitem not at all important	○	○	◉	○	○	essential

Selectie wissen

Does your paper address subitem 1b-v?

Copy and paste relevant sections from the manuscript abstract (include quotes in quotation marks "like this" to indicate direct quotes from your manuscript), or elaborate on this item by providing additional information not in the ms, or briefly explain why the item is not applicable/relevant for your study

not applicable because there were positive effects

INTRODUCTION

2a) In INTRODUCTION: Scientific background and explanation of rationale

2a-i) Problem and the type of system/solution

Describe the problem and the type of system/solution that is object of the study: intended as stand-alone intervention vs. incorporated in broader health care program? Intended for a particular patient population? Goals of the intervention, e.g., being more cost-effective to other interventions, replace or complement other solutions? (Note: Details about the intervention are provided in “Methods” under 5)

	1	2	3	4	5	
subitem not at all important	○	○	○	◉	○	essential

Selectie wissen

Does your paper address subitem 2a-i? *

Copy and paste relevant sections from the manuscript (include quotes in quotation marks "like this" to indicate direct quotes from your manuscript), or elaborate on this item by providing additional information not in the ms, or briefly explain why the item is not applicable/relevant for your study

"Financial incentives are often added as a supplement to already active behavior change interventions and even then roughly double the odds of successful behavior change ([16]."

2a-ii) Scientific background, rationale: What is known about the (type of) system

Scientific background, rationale: What is known about the (type of) system that is the object of the study (be sure to discuss the use of similar systems for other conditions/diagnoses, if appropiate), motivation for the study, i.e. what are the reasons for and what is the context for this specific study, from which stakeholder viewpoint is the study performed, potential impact of findings [2]. Briefly justify the choice of the comparator.

	1	2	3	4	5	
subitem not at all important	○	○	○	○	◉	essential

Selectie wissen

Does your paper address subitem 2a-ii? *

Copy and paste relevant sections from the manuscript (include quotes in quotation marks "like this" to indicate direct quotes from your manuscript), or elaborate on this item by providing additional information not in the ms, or briefly explain why the item is not applicable/relevant for your study

"Existing evidence indicates that deposit contracts are effective to help people lose weight [27], stop smoking [20,28] and increase physical activity [21,22,25,29–31]. A concern, however, is that voluntary uptake of deposit contracts is generally low [20,32]. In fact, some authors suggest that those who would benefit most from interventions using incentives with potential losses are not likely to enter into them[33,34]. Yet, comparing evidence on the uptake and effectiveness of deposit contracts for physical activity between studies is complicated, as operationalisations differ substantially. In particular, three different types of deposit contracts can be distinguished. First, in line with their potential to promote cost-sharing, several authors have used completely self-funded deposit contracts [32,35]. Without potential for financial gain, such self-funded deposit contracts involve only losses compared to status quo. Second, uptake of deposit contracts is often encouraged through 'matching' individuals' contribution into the deposit scheme or combining deposits with a reward-based incentive [20,36,37]. Such matched deposit contracts, thus, involve both potential gains and losses compared to status quo. Third, some authors have used loss framing to mimic the feelings of loss involved in a deposit contract, without actually requiring individuals to put their own money at risk [21,25]. For example, in a loss framed condition, Patel et al. [21] promised respondents $42 upfront of which they could then lose $1.40 for every day they did not attain physical activity goals. This loss framed condition proved more effective in promoting physical activity compared to a gain framed condition in which respondents simply earned $1.40 for every day they did attain physical activity goals. However, participants in all conditions of this study faced no actual losses, but in fact were making gains compared to their pre-intervention status quo.

The current study
In this study, we investigate the impact of deposit contracts on increasing physical activity by disentangling the effects of incurring actual losses (through self-funding) and loss framing."

2b) In INTRODUCTION: Specific objectives or hypotheses

Does your paper address CONSORT subitem 2b? *

Copy and paste relevant sections from the manuscript (include quotes in quotation marks "like this" to indicate direct quotes from your manuscript), or elaborate on this item by providing additional information not in the ms, or briefly explain why the item is not applicable/relevant for your study

"We will employ an actual deposit contract (i.e. a stick) that requires participants to make a deposit of their own money before the intervention starts, and compare this with receiving a reward (i.e. a carrot) of equivalent size. In line with Adams et al. [38] we will refer to this as the direction of incentives. Furthermore, we will investigate if loss framing (compared to gain framing) enhances the effectiveness of both reward and deposit contract incentives. Firstly, we expect that, overall, incentive conditions are more effective than an active no-incentive control condition (H1). Furthermore, we hypothesize that deposit contracts will have lower uptake than regular rewards (H2) yet, deposit contracts are expected to be more effective than regular rewards for those that partake in the intervention (H3). Additionally, we hypothesize that loss framing an incentive will increase effectiveness compared to gain framing (H4). Finally, we propose that incentives in which both direction of the incentive and framing of the incentive are loss-congruent (i.e., loss framed deposit contracts), are most likely to invoke loss aversion and therefore especially effective in promoting physical activity (H5). "

METHODS

3a) Description of trial design (such as parallel, factorial) including allocation ratio

Does your paper address CONSORT subitem 3a? *

Copy and paste relevant sections from the manuscript (include quotes in quotation marks "like this" to indicate direct quotes from your manuscript), or elaborate on this item by providing additional information not in the ms, or briefly explain why the item is not applicable/relevant for your study

"We employed a 2: incentive direction (reward/deposit) x 2: feedback frame (gain/loss) design with an additional control condition. Participants were automatically randomized over these five conditions by the app. "

3b) Important changes to methods after trial commencement (such as eligibility criteria), with reasons

Does your paper address CONSORT subitem 3b? *

Copy and paste relevant sections from the manuscript (include quotes in quotation marks "like this" to indicate direct quotes from your manuscript), or elaborate on this item by providing additional information not in the ms, or briefly explain why the item is not applicable/relevant for your study

N/A because we did not make changes

3b-i) Bug fixes, Downtimes, Content Changes

Bug fixes, Downtimes, Content Changes: ehealth systems are often dynamic systems. A description of changes to methods therefore also includes important changes made on the intervention or comparator during the trial (e.g., major bug fixes or changes in the functionality or content) (5-iii) and other “unexpected events” that may have influenced study design such as staff changes, system failures/downtimes, etc. [2].

	1	2	3	4	5	
subitem not at all important	○	○	○	○	○	essential

Does your paper address subitem 3b-i?

Copy and paste relevant sections from the manuscript (include quotes in quotation marks "like this" to indicate direct quotes from your manuscript), or elaborate on this item by providing additional information not in the ms, or briefly explain why the item is not applicable/relevant for your study

N/A because there was no downtime or changes made

4a) Eligibility criteria for participants

Does your paper address CONSORT subitem 4a? *

Copy and paste relevant sections from the manuscript (include quotes in quotation marks "like this" to indicate direct quotes from your manuscript), or elaborate on this item by providing additional information not in the ms, or briefly explain why the item is not applicable/relevant for your study

"We recruited healthy participants between 18 and 30 years old through a university research participation system (SONA), flyers on campus and posts on social media. Participants had to be willing to improve their physical activity, own a smartphone and be proficient in English."

4a-i) Computer / Internet literacy

Computer / Internet literacy is often an implicit “de facto” eligibility criterion - this should be explicitly clarified.

	1	2	3	4	5	
subitem not at all important	○	○	●	○	○	essential

Selectie wissen

Does your paper address subitem 4a-i?

Copy and paste relevant sections from the manuscript (include quotes in quotation marks "like this" to indicate direct quotes from your manuscript), or elaborate on this item by providing additional information not in the ms, or briefly explain why the item is not applicable/relevant for your study

N/A since all participants had to own a smartphone we considered them to be literate enough

4a-ii) Open vs. closed, web-based vs. face-to-face assessments:

Open vs. closed, web-based vs. face-to-face assessments: Mention how participants were recruited (online vs. offline), e.g., from an open access website or from a clinic, and clarify if this was a purely web-based trial, or there were face-to-face components (as part of the intervention or for assessment), i.e., to what degree got the study team to know the participant. In online-only trials, clarify if participants were quasi-anonymous and whether having multiple identities was possible or whether technical or logistical measures (e.g., cookies, email confirmation, phone calls) were used to detect/prevent these.

	1	2	3	4	5	
subitem not at all important	○	○	○	○	◉	essential

Selectie wissen

Does your paper address subitem 4a-ii? *

Copy and paste relevant sections from the manuscript (include quotes in quotation marks "like this" to indicate direct quotes from your manuscript), or elaborate on this item by providing additional information not in the ms, or briefly explain why the item is not applicable/relevant for your study

Registration of e-mail addresses was used to determine duplicate accounts which were removed from analysis

4a-iii) Information giving during recruitment

Information given during recruitment. Specify how participants were briefed for recruitment and in the informed consent procedures (e.g., publish the informed consent documentation as appendix, see also item X26), as this information may have an effect on user self-selection, user expectation and may also bias results.

	1	2	3	4	5	
subitem not at all important	○	○	○	○	◉	essential

Selectie wissen

Does your paper address subitem 4a-iii?

Copy and paste relevant sections from the manuscript (include quotes in quotation marks "like this" to indicate direct quotes from your manuscript), or elaborate on this item by providing additional information not in the ms, or briefly explain why the item is not applicable/relevant for your study

"We obtained informed consent before the start of the study"

4b) Settings and locations where the data were collected

Does your paper address CONSORT subitem 4b? *

Copy and paste relevant sections from the manuscript (include quotes in quotation marks "like this" to indicate direct quotes from your manuscript), or elaborate on this item by providing additional information not in the ms, or briefly explain why the item is not applicable/relevant for your study

N/A study was performed fully online

4b-i) Report if outcomes were (self-)assessed through online questionnaires

Clearly report if outcomes were (self-)assessed through online questionnaires (as common in web-based trials) or otherwise.

	1	2	3	4	5	
subitem not at all important	○	○	○	○	●	essential

Selectie wissen

Does your paper address subitem 4b-i? *

Copy and paste relevant sections from the manuscript (include quotes in quotation marks "like this" to indicate direct quotes from your manuscript), or elaborate on this item by providing additional information not in the ms, or briefly explain why the item is not applicable/relevant for your study

N/A primary and secondary outcomes were automatically registered

4b-ii) Report how institutional affiliations are displayed

Report how institutional affiliations are displayed to potential participants [on ehealth media], as affiliations with prestigious hospitals or universities may affect volunteer rates, use, and reactions with regards to an intervention.(Not a required item – describe only if this may bias results)

	1	2	3	4	5	
subitem not at all important	○	○	◉	○	○	essential

Selectie wissen

Does your paper address subitem 4b-ii?

Copy and paste relevant sections from the manuscript (include quotes in quotation marks "like this" to indicate direct quotes from your manuscript), or elaborate on this item by providing additional information not in the ms, or briefly explain why the item is not applicable/relevant for your study

No. Leiden University logo was displayed when downloading and installing the application

5) The interventions for each group with sufficient details to allow replication, including how and when they were actually administered

5-i) Mention names, credential, affiliations of the developers, sponsors, and owners

Mention names, credential, affiliations of the developers, sponsors, and owners [6] (if authors/evaluators are owners or developer of the software, this needs to be declared in a “Conflict of interest” section or mentioned elsewhere in the manuscript).

	1	2	3	4	5	
subitem not at all important	○	○	○	○	●	essential

Selectie wissen

Does your paper address subitem 5-i?

Copy and paste relevant sections from the manuscript (include quotes in quotation marks "like this" to indicate direct quotes from your manuscript), or elaborate on this item by providing additional information not in the ms, or briefly explain why the item is not applicable/relevant for your study

"The intervention for this study was delivered entirely online via the Benefit Move application which participants downloaded on their smartphones. The Benefit Move application was implemented using MobileCoach [42,43], an open-source software platform for smartphone-based and chatbot-delivered behavioral interventions (e.g. [44]) and ecological momentary assessments (e.g. [45]). MobileCoach was developed by the Centre for Digital Health Interventions (CDHI) at ETH Zurich & University of St. Gallen in Switzerland (www.mobile-coach.eu). The Benefit Move application had two main functions: (1) objectively measuring physical activity and (2) communicating with the participant. " and "Conflicts of Interest
PS and TK are affiliated with the Centre for Digital Health Interventions, a joint initiative of the Department of Management, Technology, and Economics at ETH Zurich and the Institute of Technology Management at the University of St Gallen, which is funded in part by the Swiss health insurer, CSS. TK is also cofounder of Pathmate Technologies, a university spin-off company that creates and delivers digital clinical pathways. However, neither CSS nor Pathmate Technologies was involved in this study. "

5-ii) Describe the history/development process

Describe the history/development process of the application and previous formative evaluations (e.g., focus groups, usability testing), as these will have an impact on adoption/use rates and help with interpreting results.

	1	2	3	4	5	
subitem not at all important	○	○	○	○	◉	essential

Selectie wissen

Does your paper address subitem 5-ii?

Copy and paste relevant sections from the manuscript (include quotes in quotation marks "like this" to indicate direct quotes from your manuscript), or elaborate on this item by providing additional information not in the ms, or briefly explain why the item is not applicable/relevant for your study

N/A we developed the intervention specifically for this study so no version updates or evaluation have taken place prior

5-iii) Revisions and updating

Revisions and updating. Clearly mention the date and/or version number of the application/intervention (and comparator, if applicable) evaluated, or describe whether the intervention underwent major changes during the evaluation process, or whether the development and/or content was "frozen" during the trial. Describe dynamic components such as news feeds or changing content which may have an impact on the replicability of the intervention (for unexpected events see item 3b).

	1	2	3	4	5	
subitem not at all important	○	○	○	○	◉	essential

Selectie wissen

Does your paper address subitem 5-iii?

Copy and paste relevant sections from the manuscript (include quotes in quotation marks "like this" to indicate direct quotes from your manuscript), or elaborate on this item by providing additional information not in the ms, or briefly explain why the item is not applicable/relevant for your study

N/A no changes or updates were done during the intervention period

5-iv) Quality assurance methods

Provide information on quality assurance methods to ensure accuracy and quality of information provided [1], if applicable.

	1	2	3	4	5	
subitem not at all important	○	○	●	○	○	essential

Selectie wissen

Does your paper address subitem 5-iv?

Copy and paste relevant sections from the manuscript (include quotes in quotation marks "like this" to indicate direct quotes from your manuscript), or elaborate on this item by providing additional information not in the ms, or briefly explain why the item is not applicable/relevant for your study

N/A since primary outcomes were automatically registered this was not considered crucial

5-v) Ensure replicability by publishing the source code, and/or providing screenshots/screen-capture video, and/or providing flowcharts of the algorithms used

Ensure replicability by publishing the source code, and/or providing screenshots/screen-capture video, and/or providing flowcharts of the algorithms used. Replicability (i.e., other researchers should in principle be able to replicate the study) is a hallmark of scientific reporting.

	1	2	3	4	5	
subitem not at all important	○	○	○	◉	○	essential

Selectie wissen

Does your paper address subitem 5-v?

Copy and paste relevant sections from the manuscript (include quotes in quotation marks "like this" to indicate direct quotes from your manuscript), or elaborate on this item by providing additional information not in the ms, or briefly explain why the item is not applicable/relevant for your study

N/A since the app was built on an open-source platform, others can use the same functionality

5-vi) Digital preservation

Digital preservation: Provide the URL of the application, but as the intervention is likely to change or disappear over the course of the years; also make sure the intervention is archived (Internet Archive, webcitation.org, and/or publishing the source code or screenshots/videos alongside the article). As pages behind login screens cannot be archived, consider creating demo pages which are accessible without login.

	1	2	3	4	5	
subitem not at all important	○	◉	○	○	○	essential

Selectie wissen

Does your paper address subitem 5-vi?

Copy and paste relevant sections from the manuscript (include quotes in quotation marks "like this" to indicate direct quotes from your manuscript), or elaborate on this item by providing additional information not in the ms, or briefly explain why the item is not applicable/relevant for your study

The app was built on an open-source platform and the developers have access to the code

5-vii) Access

Access: Describe how participants accessed the application, in what setting/context, if they had to pay (or were paid) or not, whether they had to be a member of specific group. If known, describe how participants obtained "access to the platform and Internet" [1]. To ensure access for editors/reviewers/readers, consider to provide a "backdoor" login account or demo mode for reviewers/readers to explore the application (also important for archiving purposes, see vi).

	1	2	3	4	5	
subitem not at all important	○	○	○	●	○	essential

Selectie wissen

Does your paper address subitem 5-vii? *

Copy and paste relevant sections from the manuscript (include quotes in quotation marks "like this" to indicate direct quotes from your manuscript), or elaborate on this item by providing additional information not in the ms, or briefly explain why the item is not applicable/relevant for your study

"One week prior to the start of the intervention, participants filled in the screening survey with inclusion and exclusion criteria and provided digital informed consent. Thereafter, eligible participants received an URL to the iOS or Android app stores where they could download the Benefit Move intervention application and install it on their smartphone. Once participants installed the app, they were asked to complete onboarding in the app within two days. Thereafter, participants were sent a link to the survey platform LimeSurvey [49] that opened within the Benefit Move app. Here, they filled in the baseline survey (for more detail see baseline survey) and then returned to the app after completion. Participants were excluded from the study if they did not complete the onboarding and baseline survey before the start of the intervention. "

5-viii) Mode of delivery, features/functionalities/components of the intervention and comparator, and the theoretical framework

Describe mode of delivery, features/functionalities/components of the intervention and comparator, and the theoretical framework [6] used to design them (instructional strategy [1], behaviour change techniques, persuasive features, etc., see e.g., [7, 8] for terminology). This includes an in-depth description of the content (including where it is coming from and who developed it) [1]," whether [and how] it is tailored to individual circumstances and allows users to track their progress and receive feedback" [6]. This also includes a description of communication delivery channels and – if computer-mediated communication is a component – whether communication was synchronous or asynchronous [6]. It also includes information on presentation strategies [1], including page design principles, average amount of text on pages, presence of hyperlinks to other resources, etc. [1].

	1	2	3	4	5	
subitem not at all important	○	○	○	○	◉	essential

Selectie wissen

Does your paper address subitem 5-viii? *

Copy and paste relevant sections from the manuscript (include quotes in quotation marks "like this" to indicate direct quotes from your manuscript), or elaborate on this item by providing additional information not in the ms, or briefly explain why the item is not applicable/relevant for your study

"After participants completed the baseline survey, they received a tailored step goal based on their 7-day historic daily step average that was retrieved through Google Fit or Apple Health. Retrieving step counts for 7 consecutive days should accurately estimate habitual activity levels of individuals [50], and providing an individualised and realistic goal should increase intervention effectiveness [15]. If historic data was available, the participant was assigned a goal that was 120% of the historic daily step average. For example, someone who, in the 7 days prior to goal setting, took on average 5000 steps per day would automatically receive a 6000 steps daily step goal. If no historic data was available, the participant was assigned a default step goal of 10.000 steps per day because that is an often-used guideline for sufficient physical activity [51].
All participants started simultaneously with the 20-day intervention on Monday March 20th 2020 at 09.00 (9 AM). Each day during the 20-day intervention participants received a push notification at 09.00 in the morning (9 AM). This notification prompted them to click a button to retrieve their step count performance of the past day and get an update on the progress for the current day. If the user skipped doing this for several days, but then responded and requested an update, the feedback for multiple days was given in separate consecutive messages, with a separate update message per day. The feedback per day consisted of: the achieved step count compared to the daily step goal, a conclusion about whether the goal was achieved or not, the money that was earned or lost on that day, and the running total of earnings or losses during the entire intervention (see Figure 1 for an example). Based on their condition, participants received different instructions at the start of the intervention and received different feedback messages during the intervention.
"

5-ix) Describe use parameters

Describe use parameters (e.g., intended “doses” and optimal timing for use). Clarify what instructions or recommendations were given to the user, e.g., regarding timing, frequency, heaviness of use, if any, or was the intervention used ad libitum.

	1	2	3	4	5	
subitem not at all important	○	○	○	○	◉	essential

Selectie wissen

Does your paper address subitem 5-ix?

Copy and paste relevant sections from the manuscript (include quotes in quotation marks "like this" to indicate direct quotes from your manuscript), or elaborate on this item by providing additional information not in the ms, or briefly explain why the item is not applicable/relevant for your study

"Each day during the 20-day intervention participants received a push notification at 09.00 in the morning (9 AM). This notification prompted them to click a button to retrieve their step count performance of the past day and get an update on the progress for the current day. If the user skipped doing this for several days, but then responded and requested an update, the feedback for multiple days was given in separate consecutive messages, with a separate update message per day. The feedback per day consisted of: the achieved step count compared to the daily step goal, a conclusion about whether the goal was achieved or not, the money that was earned or lost on that day, and the running total of earnings or losses during the entire intervention (see Figure 1 for an example)."

5-x) Clarify the level of human involvement

Clarify the level of human involvement (care providers or health professionals, also technical assistance) in the e-intervention or as co-intervention (detail number and expertise of professionals involved, if any, as well as “type of assistance offered, the timing and frequency of the support, how it is initiated, and the medium by which the assistance is delivered”. It may be necessary to distinguish between the level of human involvement required for the trial, and the level of human involvement required for a routine application outside of a RCT setting (discuss under item 21 – generalizability).

	1	2	3	4	5	
subitem not at all important	○	○	○	●	○	essential

Selectie wissen

Does your paper address subitem 5-x?

Copy and paste relevant sections from the manuscript (include quotes in quotation marks "like this" to indicate direct quotes from your manuscript), or elaborate on this item by providing additional information not in the ms, or briefly explain why the item is not applicable/relevant for your study

The intervention was delivered fully online

5-xi) Report any prompts/reminders used

Report any prompts/reminders used: Clarify if there were prompts (letters, emails, phone calls, SMS) to use the application, what triggered them, frequency etc. It may be necessary to distinguish between the level of prompts/reminders required for the trial, and the level of prompts/reminders for a routine application outside of a RCT setting (discuss under item 21 – generalizability).

	1	2	3	4	5	
subitem not at all important	○	○	○	○	◉	essential

Selectie wissen

Does your paper address subitem 5-xi? *

Copy and paste relevant sections from the manuscript (include quotes in quotation marks "like this" to indicate direct quotes from your manuscript), or elaborate on this item by providing additional information not in the ms, or briefly explain why the item is not applicable/relevant for your study

We used prompts to get participants to pay the deposit for the study. See manuscript: "If no payment was received, participants were automatically reminded via push message, text message, telephone and e-mail reminders. Participants were excluded if deposit payments were not confirmed 12 hours before the start of the intervention. After we confirmed the received deposit payment, participants were instructed to wait until the intervention started the next Monday morning."

5-xii) Describe any co-interventions (incl. training/support)

Describe any co-interventions (incl. training/support): Clearly state any interventions that are provided in addition to the targeted eHealth intervention, as ehealth intervention may not be designed as stand-alone intervention. This includes training sessions and support [1]. It may be necessary to distinguish between the level of training required for the trial, and the level of training for a routine application outside of a RCT setting (discuss under item 21 – generalizability.

	1	2	3	4	5	
subitem not at all important	○	○	○	◉	○	essential

Selectie wissen

Does your paper address subitem 5-xii? *

Copy and paste relevant sections from the manuscript (include quotes in quotation marks "like this" to indicate direct quotes from your manuscript), or elaborate on this item by providing additional information not in the ms, or briefly explain why the item is not applicable/relevant for your study

N/A we did not perform any co-intervention

6a) Completely defined pre-specified primary and secondary outcome measures, including how and when they were assessed

Does your paper address CONSORT subitem 6a? *

Copy and paste relevant sections from the manuscript (include quotes in quotation marks "like this" to indicate direct quotes from your manuscript), or elaborate on this item by providing additional information not in the ms, or briefly explain why the item is not applicable/relevant for your study

"The primary outcome (continuous) was effectiveness. This was measured through mobile registration of step count data and defined as the number of days (0-20) the goal was achieved. The secondary outcome (binary) was uptake of the intervention and defined as explicitly agreeing to participate in the challenge and paying the deposit (if required). "

6a-i) Online questionnaires: describe if they were validated for online use and apply CHERRIES items to describe how the questionnaires were designed/deployed

If outcomes were obtained through online questionnaires, describe if they were validated for online use and apply CHERRIES items to describe how the questionnaires were designed/deployed [9].

	1	2	3	4	5	
subitem not at all important	○	○	○	◉	○	essential

Selectie wissen

Does your paper address subitem 6a-i?

Copy and paste relevant sections from manuscript text

N/A we did not do this

Your answer must have a minimum of 25 characters.

6a-ii) Describe whether and how “use” (including intensity of use/dosage) was defined/measured/monitored

Describe whether and how “use” (including intensity of use/dosage) was defined/measured/monitored (logins, logfile analysis, etc.). Use/adoption metrics are important process outcomes that should be reported in any ehealth trial.

	1	2	3	4	5	
subitem not at all important	○	○	○	○	●	essential

Selectie wissen

Does your paper address subitem 6a-ii?

Copy and paste relevant sections from manuscript text

we analysed the number of days for which step count data had been retrieved for participants

6a-iii) Describe whether, how, and when qualitative feedback from participants was obtained

Describe whether, how, and when qualitative feedback from participants was obtained (e.g., through emails, feedback forms, interviews, focus groups).

	1	2	3	4	5	
subitem not at all important	○	○	○	○	◉	essential

Selectie wissen

Does your paper address subitem 6a-iii?

Copy and paste relevant sections from manuscript text

We did not do obtain qualitative feedback

6b) Any changes to trial outcomes after the trial commenced, with reasons

Does your paper address CONSORT subitem 6b? *

Copy and paste relevant sections from the manuscript (include quotes in quotation marks "like this" to indicate direct quotes from your manuscript), or elaborate on this item by providing additional information not in the ms, or briefly explain why the item is not applicable/relevant for your study

N/A because no changes were made

7a) How sample size was determined

NPT: When applicable, details of whether and how the clustering by care provides or centers was addressed

7a-i) Describe whether and how expected attrition was taken into account when calculating the sample size

Describe whether and how expected attrition was taken into account when calculating the sample size.

	1	2	3	4	5	
subitem not at all important	○	○	○	○	◉	essential

Selectie wissen

Does your paper address subitem 7a-i?

Copy and paste relevant sections from manuscript title (include quotes in quotation marks "like this" to indicate direct quotes from your manuscript), or elaborate on this item by providing additional information not in the ms, or briefly explain why the item is not applicable/relevant for your study

"A priori sample size calculations with G*Power [39] suggested a minimum sample size of 199 for detecting a between conditions difference in effectiveness with a medium effect size (f = .20), 80% power and an alpha of .05 (ANCOVA with 5 groups). Based on similar research [40] that showed relatively high dropout between recruitment and participation, we assumed dropout of 20% and aimed to recruit 240 eligible participants. "

7b) When applicable, explanation of any interim analyses and stopping guidelines

Does your paper address CONSORT subitem 7b? *

Copy and paste relevant sections from the manuscript (include quotes in quotation marks "like this" to indicate direct quotes from your manuscript), or elaborate on this item by providing additional information not in the ms, or briefly explain why the item is not applicable/relevant for your study

N/A other than reported in power analysis

8a) Method used to generate the random allocation sequence

NPT: When applicable, how care providers were allocated to each trial group

Does your paper address CONSORT subitem 8a? *

Copy and paste relevant sections from the manuscript (include quotes in quotation marks "like this" to indicate direct quotes from your manuscript), or elaborate on this item by providing additional information not in the ms, or briefly explain why the item is not applicable/relevant for your study

" Participants were automatically randomized over these five conditions by the app. "

8b) Type of randomisation; details of any restriction (such as blocking and block size)

Does your paper address CONSORT subitem 8b? *

Copy and paste relevant sections from the manuscript (include quotes in quotation marks "like this" to indicate direct quotes from your manuscript), or elaborate on this item by providing additional information not in the ms, or briefly explain why the item is not applicable/relevant for your study

N/A because participants were automatically and equally randomised over conditions

9) Mechanism used to implement the random allocation sequence (such as sequentially numbered containers), describing any steps taken to conceal the sequence until interventions were assigned

Does your paper address CONSORT subitem 9? *

Copy and paste relevant sections from the manuscript (include quotes in quotation marks "like this" to indicate direct quotes from your manuscript), or elaborate on this item by providing additional information not in the ms, or briefly explain why the item is not applicable/relevant for your study

N/A because participants were automatically and equally randomised over conditions

10) Who generated the random allocation sequence, who enrolled participants, and who assigned participants to interventions

Does your paper address CONSORT subitem 10? *

Copy and paste relevant sections from the manuscript (include quotes in quotation marks "like this" to indicate direct quotes from your manuscript), or elaborate on this item by providing additional information not in the ms, or briefly explain why the item is not applicable/relevant for your study

N/A because participants were automatically and equally randomised over conditions

11a) If done, who was blinded after assignment to interventions (for example, participants, care providers, those assessing outcomes) and how

NPT: Whether or not administering co-interventions were blinded to group assignment

11a-i) Specify who was blinded, and who wasn’t

Specify who was blinded, and who wasn’t. Usually, in web-based trials it is not possible to blind the participants [1, 3] (this should be clearly acknowledged), but it may be possible to blind outcome assessors, those doing data analysis or those administering co-interventions (if any).

	1	2	3	4	5	
subitem not at all important	○	○	○	◉	○	essential

Selectie wissen

Does your paper address subitem 11a-i? *

Copy and paste relevant sections from the manuscript (include quotes in quotation marks "like this" to indicate direct quotes from your manuscript), or elaborate on this item by providing additional information not in the ms, or briefly explain why the item is not applicable/relevant for your study

N/A because participants were automatically and equally randomised over conditions and the intervention was delivered fully automatic and online without researcher involvement

11a-ii) Discuss e.g., whether participants knew which intervention was the “intervention of interest” and which one was the “comparator”

Informed consent procedures (4a-ii) can create biases and certain expectations - discuss e.g., whether participants knew which intervention was the “intervention of interest” and which one was the “comparator”.

	1	2	3	4	5	
subitem not at all important	○	○	○	○	◉	essential

Selectie wissen

Does your paper address subitem 11a-ii?

Copy and paste relevant sections from the manuscript (include quotes in quotation marks "like this" to indicate direct quotes from your manuscript), or elaborate on this item by providing additional information not in the ms, or briefly explain why the item is not applicable/relevant for your study

"Furthermore, before onboarding, participants read the informed consent which mentioned that the study possibly required them to deposit € 10 of their own money. Mentioning this possibility was important for informed consent, but may have deterred some participants from participating before they onboarded in the application. It is possible that this biased our analysis of uptake, and that the actual uptake of deposit contracts is lower than our analyses suggest."

11b) If relevant, description of the similarity of interventions

(this item is usually not relevant for ehealth trials as it refers to similarity of a placebo or sham intervention to a active medication/intervention)

Does your paper address CONSORT subitem 11b? *

Copy and paste relevant sections from the manuscript (include quotes in quotation marks "like this" to indicate direct quotes from your manuscript), or elaborate on this item by providing additional information not in the ms, or briefly explain why the item is not applicable/relevant for your study

N/A because no placebo intervention

12a) Statistical methods used to compare groups for primary and secondary outcomes

NPT: When applicable, details of whether and how the clustering by care providers or centers was addressed

Does your paper address CONSORT subitem 12a? *

Copy and paste relevant sections from the manuscript (include quotes in quotation marks "like this" to indicate direct quotes from your manuscript), or elaborate on this item by providing additional information not in the ms, or briefly explain why the item is not applicable/relevant for your study

"Hypothesis 1: Effectiveness of incentive conditions compared to control condition. Firstly, we performed an ANCOVA with baseline steps as a covariate in which we compiled across incentive conditions to compare all incentive conditions combined (mean of conditions 2-5) to the control condition (i.e., condition 1). Secondly, we performed an ANCOVA with baseline steps as a covariate and effectiveness as the dependent variable to separately compare incentive conditions (i.e., conditions 2-5) to the no-incentive control condition (i.e., condition 1). The ANCOVA was performed with factor 'condition' with 5 levels (condition 1, 2, 3, 4, and 5). We compared every incentive group separately to the control condition with 4 planned contrasts: 1 = control vs deposit/gain, 2 = control vs deposit/loss, 3 = control vs reward/gain, 4 = control vs reward/loss.
Hypothesis 2: Uptake of the intervention. We performed a Chi-square test of independence to investigate whether the uptake was lower for deposit contracts (i.e., condition 4 & 5) compared to regular rewards (i.e., condition 2 & 3).
Hypothesis 3-5: The effect of incentive direction and feedback framing on effectiveness. We performed a two-way ANCOVA with baseline steps as a covariate. Effectiveness was the dependent variable and the model contained two factors: incentive direction (deposit/reward) and feedback frame (loss/gain). In the model, we specified both main effects of the factors (H2-H3) and their interaction (H4)."

12a-i) Imputation techniques to deal with attrition / missing values

Imputation techniques to deal with attrition / missing values: Not all participants will use the intervention/comparator as intended and attrition is typically high in ehealth trials. Specify how participants who did not use the application or dropped out from the trial were treated in the statistical analysis (a complete case analysis is strongly discouraged, and simple imputation techniques such as LOCF may also be problematic [4]).

	1	2	3	4	5	
subitem not at all important	○	○	○	○	◉	essential

Selectie wissen

Does your paper address subitem 12a-i? *

Copy and paste relevant sections from the manuscript (include quotes in quotation marks "like this" to indicate direct quotes from your manuscript), or elaborate on this item by providing additional information not in the ms, or briefly explain why the item is not applicable/relevant for your study

"We dealt with missing cases by using pairwise exclusion and used standard p <.05 criterium for determining statistical significance"

12b) Methods for additional analyses, such as subgroup analyses and adjusted analyses

Does your paper address CONSORT subitem 12b? *

Copy and paste relevant sections from the manuscript (include quotes in quotation marks "like this" to indicate direct quotes from your manuscript), or elaborate on this item by providing additional information not in the ms, or briefly explain why the item is not applicable/relevant for your study

"We report results on effectiveness based on a restricted sample that only included participants who retrieved steps on at least one intervention day and who received a tailored step goal. We excluded participants that received a default goal, because in hindsight these participants were confronted with a goal that was unachievable (see Appendix C for an overview of analyses where these participants are included). Furthermore, we report the main analyses for effectiveness based on models that include baseline step counts as a covariate. The pattern of results was similar, but the models gained in accuracy by including the covariate."

X26) REB/IRB Approval and Ethical Considerations [recommended as subheading under "Methods"] (not a CONSORT item)

X26-i) Comment on ethics committee approval

	1	2	3	4	5	
subitem not at all important	○	○	○	○	◉	essential

Selectie wissen

Does your paper address subitem X26-i?

Copy and paste relevant sections from the manuscript (include quotes in quotation marks "like this" to indicate direct quotes from your manuscript), or elaborate on this item by providing additional information not in the ms, or briefly explain why the item is not applicable/relevant for your study

"The study was approved by the Psychology Research Ethics Committee of Leiden University (2020-02-24-T. Reijnders-V2-2089) and the study protocol was preregistered on Open Science Framework (https://osf.io/34ygt). "

x26-ii) Outline informed consent procedures

Outline informed consent procedures e.g., if consent was obtained offline or online (how? Checkbox, etc.?), and what information was provided (see 4a-ii). See [6] for some items to be included in informed consent documents.

	1	2	3	4	5	
subitem not at all important	○	○	○	◉	○	essential

Selectie wissen

Does your paper address subitem X26-ii?

Copy and paste relevant sections from the manuscript (include quotes in quotation marks "like this" to indicate direct quotes from your manuscript), or elaborate on this item by providing additional information not in the ms, or briefly explain why the item is not applicable/relevant for your study

Not included in MS. Informed consent was obtained online prior to the study

X26-iii) Safety and security procedures

Safety and security procedures, incl. privacy considerations, and any steps taken to reduce the likelihood or detection of harm (e.g., education and training, availability of a hotline)

	1	2	3	4	5	
subitem not at all important	○	○	○	●	○	essential

Selectie wissen

Does your paper address subitem X26-iii?

Copy and paste relevant sections from the manuscript (include quotes in quotation marks "like this" to indicate direct quotes from your manuscript), or elaborate on this item by providing additional information not in the ms, or briefly explain why the item is not applicable/relevant for your study

Not in MS because not considered crucial for the MS

RESULTS

13a) For each group, the numbers of participants who were randomly assigned, received intended treatment, and were analysed for the primary outcome

NPT: The number of care providers or centers performing the intervention in each group and the number of patients treated by each care provider in each center

Does your paper address CONSORT subitem 13a? *

Copy and paste relevant sections from the manuscript (include quotes in quotation marks "like this" to indicate direct quotes from your manuscript), or elaborate on this item by providing additional information not in the ms, or briefly explain why the item is not applicable/relevant for your study

This is included in table 2 in the MS. See:

"Table 2. Descriptive overview of results (N = 126)

Variable	Condition					
	Control	Reward / Gain frame	Reward / Loss frame	Deposit / Gain frame	Deposit / Loss frame	Total
N	29	32	18	23	24	126
Uptake	29 (100%)	32 (100%)	18 (100%)	15 (65%)	14 (58%)	108 (86%)
Explicit refusal	0	0	0	4	7	11
Deposit not paid	-	-	-	4	3	7
Never retrieved steps	2	4	3	0	3	12
Goal type						
Tailored goals	18 (62%)	21 (66%)	11 (61%)	17 (74%)	14 (58%)	81 (68%)
Default goals 10K	11 (38 %)	11 (34%)	7 (39%)	6 (26%)	10 (42%)	45 (36%)

"

13b) For each group, losses and exclusions after randomisation, together with reasons

Does your paper address CONSORT subitem 13b? (NOTE: Preferably, this is shown in a CONSORT flow diagram) *

Copy and paste relevant sections from the manuscript (include quotes in quotation marks "like this" to indicate direct quotes from your manuscript), or elaborate on this item by providing additional information not in the ms, or briefly explain why the item is not applicable/relevant for your study

This is shown in CONSORT flow diagram in appendix of MS

13b-i) Attrition diagram

Strongly recommended: An attrition diagram (e.g., proportion of participants still logging in or using the intervention/comparator in each group plotted over time, similar to a survival curve) or other figures or tables demonstrating usage/dose/engagement.

	1	2	3	4	5	
subitem not at all important	○	○	○	○	○	essential

Does your paper address subitem 13b-i?

Copy and paste relevant sections from the manuscript or cite the figure number if applicable (include quotes in quotation marks "like this" to indicate direct quotes from your manuscript), or elaborate on this item by providing additional information not in the ms, or briefly explain why the item is not applicable/relevant for your study

This is shown in CONSORT flow diagram in appendix of MS

14a) Dates defining the periods of recruitment and follow-up

Does your paper address CONSORT subitem 14a? *

Copy and paste relevant sections from the manuscript (include quotes in quotation marks "like this" to indicate direct quotes from your manuscript), or elaborate on this item by providing additional information not in the ms, or briefly explain why the item is not applicable/relevant for your study

Did not include this in MS because not considered crucial

14a-i) Indicate if critical “secular events” fell into the study period

Indicate if critical “secular events” fell into the study period, e.g., significant changes in Internet resources available or “changes in computer hardware or Internet delivery resources”

	1	2	3	4	5	
subitem not at all important	○	○	○	○	◉	essential

Selectie wissen

Does your paper address subitem 14a-i?

Copy and paste relevant sections from the manuscript (include quotes in quotation marks "like this" to indicate direct quotes from your manuscript), or elaborate on this item by providing additional information not in the ms, or briefly explain why the item is not applicable/relevant for your study

In appendix we report sensitive checks including secular events related to COVID. See: "We performed several additional checks to test the sensitivity of the main findings to possible cheating, anxiety symptoms (related to the COVID-19 lockdown), experienced COVID-19 symptoms, whether participants were less active due to COVID-19, contamination between conditions and whether people carried their smartphone more often."

14b) Why the trial ended or was stopped (early)

Does your paper address CONSORT subitem 14b? *

Copy and paste relevant sections from the manuscript (include quotes in quotation marks "like this" to indicate direct quotes from your manuscript), or elaborate on this item by providing additional information not in the ms, or briefly explain why the item is not applicable/relevant for your study

N/A because it ran for the full period

15) A table showing baseline demographic and clinical characteristics for each group

NPT: When applicable, a description of care providers (case volume, qualification, expertise, etc.) and centers (volume) in each group

Does your paper address CONSORT subitem 15? *

Copy and paste relevant sections from the manuscript (include quotes in quotation marks "like this" to indicate direct quotes from your manuscript), or elaborate on this item by providing additional information not in the ms, or briefly explain why the item is not applicable/relevant for your study

Included in MS in table 1, but not separate per group because this was not considered crucial

15-i) Report demographics associated with digital divide issues

In ehealth trials it is particularly important to report demographics associated with digital divide issues, such as age, education, gender, social-economic status, computer/Internet/ehealth literacy of the participants, if known.

	1	2	3	4	5	
subitem not at all important	○	○	○	○	◉	essential

Selectie wissen

Does your paper address subitem 15-i? *

Copy and paste relevant sections from the manuscript (include quotes in quotation marks "like this" to indicate direct quotes from your manuscript), or elaborate on this item by providing additional information not in the ms, or briefly explain why the item is not applicable/relevant for your study

we report on age, gender, work status, income and weight

16) For each group, number of participants (denominator) included in each analysis and whether the analysis was by original assigned groups

16-i) Report multiple “denominators” and provide definitions

Report multiple “denominators” and provide definitions: Report N’s (and effect sizes) “across a range of study participation [and use] thresholds” [1], e.g., N exposed, N consented, N used more than x times, N used more than y weeks, N participants “used” the intervention/comparator at specific pre-defined time points of interest (in absolute and relative numbers per group). Always clearly define “use” of the intervention.

	1	2	3	4	5	
subitem not at all important	○	○	○	○	◉	essential

Selectie wissen

Does your paper address subitem 16-i? *

Copy and paste relevant sections from the manuscript (include quotes in quotation marks "like this" to indicate direct quotes from your manuscript), or elaborate on this item by providing additional information not in the ms, or briefly explain why the item is not applicable/relevant for your study

Included in table 2. See: "Table 2. Descriptive overview of results (N = 126)
Variable Condition
Control Reward / Gain frame Reward / Loss frame Deposit / Gain frame Deposit / Loss frame Total
N 29 32 18 23 24 126
Uptake 29 (100%) 32 (100%) 18 (100%) 15 (65%) 14 (58%) 108 (86%)
Explicit refusal 0 0 0 4 7 11
Deposit not paid - - - 4 3 7
Never retrieved steps 2 4 3 0 3 12
Goal type
Tailored goals 18 (62%) 21 (66%) 11 (61%) 17 (74%) 14 (58%) 81 (68%)
Default goals 10K 11 (38 %) 11 (34%) 7 (39%) 6 (26%) 10 (42%) 45 (36%)
"

16-ii) Primary analysis should be intent-to-treat

Primary analysis should be intent-to-treat, secondary analyses could include comparing only “users”, with the appropriate caveats that this is no longer a randomized sample (see 18-i).

	1	2	3	4	5	
subitem not at all important	◉	○	○	○	○	essential

Selectie wissen

Does your paper address subitem 16-ii?

Copy and paste relevant sections from the manuscript (include quotes in quotation marks "like this" to indicate direct quotes from your manuscript), or elaborate on this item by providing additional information not in the ms, or briefly explain why the item is not applicable/relevant for your study

Not performed because we considered non-users and non-uptakers as drop-out

17a) For each primary and secondary outcome, results for each group, and the estimated effect size and its precision (such as 95% confidence interval)

Does your paper address CONSORT subitem 17a? *

Copy and paste relevant sections from the manuscript (include quotes in quotation marks "like this" to indicate direct quotes from your manuscript), or elaborate on this item by providing additional information not in the ms, or briefly explain why the item is not applicable/relevant for your study

" Hypothesis 1: Effectiveness of incentive conditions compared to control condition
Firstly, a one-way ANCOVA with baseline steps as covariate showed that, overall, incentive conditions ($M = 13.10$, $SD = 6.33$) had higher effectiveness than the control condition ($M = 8.00$, $SD = 5.65$), $F(1, 62) = 10.72$, $p = .002$, $\eta p2 = .147$. Furthermore, to test specific contrasts, a second one-way ANCOVA with baseline steps as covariate showed that the factor condition was related to the effectiveness of the intervention, $F(4, 59) = 5.48$, $p = <.001$, $\eta p2 = .271$. Participants in the control condition achieved their step goal on $M = 8.00$ days ($SD = 5.65$). Planned contrasts indicate that this is significantly less than participants in the reward/gain condition ($M = 13.30$, $SD = 5.49$), $p = .003$, $SE = 1.86$. Furthermore, this is also significantly less than participants in the deposit/gain condition ($M = 17.40$, $SD = 6.17$), [illegible]

p = <.001, SE = 2.25. We did not find a significant difference between the control condition and reward/loss condition (M = 10.00, SD = 7.01), p = .232, SE = 2.19. Nor did we find a significant difference between the control condition and deposit/loss condition (M = 11.29, SD = 5.16), p = .191, SE = 2.53. Due to indications that normality of the DV was violated, we performed a Kruskall-Wallis test to check the robustness of these findings. We only found a significant contrast between the control condition and the deposit/gain condition (p = .001, adjusted with Bonferroni correction). There was no evidence of a significant difference for the other contrasts.

Hypothesis 2: Uptake of the intervention
A Chi-square test of independence showed that requiring a deposit decreased the uptake of the intervention: χ2 (1, N = 97) = 23.51, p = <.001, V = .492. In the reward conditions 100% of participants accepted the intervention, compared to 61.7% in the deposit conditions (for a descriptive overview of the results see Table 2). We explored if those with uptake differed from those with no uptake but were underpowered for these analyses and accordingly found no differences on demographics (gender, income, weight status, age) or other baseline characteristics (goal type, self-efficacy, risk proneness, self-control, autonomous motivation, extrinsic motivation, historic step count).

Table 2. Descriptive overview of results (N = 126)

Variable	Condition					
	Control	Reward / Gain frame	Reward / Loss frame	Deposit / Gain frame	Deposit / Loss frame	Total
N	29	32	18	23	24	126
Uptake	29 (100%)	32 (100%)	18 (100%)	15 (65%)	14 (58%)	108 (86%)
Explicit refusal	0	0	0	4	7	11
Deposit not paid	-	-	-	4	3	7
Never retrieved steps	2	4	3	0	3	12
Goal type						
Tailored goals	18 (62%)	21 (66%)	11 (61%)	17 (74%)	14 (58%)	81 (68%)
Default goals 10K	11 (38 %)	11 (34%)	7 (39%)	6 (26%)	10 (42%)	45 (36%)

Note: data are frequencies (%).

Hypothesis 3-4-5: The effect of incentive direction and feedback framing on effectiveness
A two-way ANCOVA with baseline steps as covariate showed no main effect of incentive direction, F(1, 43) = 1.98, p = .166, ηp2 = .044 indicating that deposits (M = 14.88, SD = 6.40) were not more effective than rewards (M = 12.13, SD = 6.17). We did find a main effect of feedback framing, F(1, 43) = 7.91, p = .007, ηp2 = .155 indicating that loss frames (M = 10.50, SD = 6.22) were significantly less effective than gain frames (M = 14.67, SD = 5.95). Finally, the interaction effect of incentive direction X feedback framing was not significant, F(1, 43) = 1.16, p = .287, ηp2 = .026 indicating that feedback framing did not have a different effect in deposit conditions compared to reward conditions. See Table 3 for a descriptive overview of the results per arm of the experiment.
Furthermore, to test the robustness of these findings, we additionally performed a Kruskall-Wallis test. For the main effects we performed two separate tests, one for each factor from [illegible]

the two-way ANOVA. The interaction effect could not be tested with this alternative method. Consistent with what was found in the two-way ANCOVA, we found that incentive direction was not significantly related to effectiveness (p = .062), but feedback framing was significantly related to effectiveness (p = .033). Additional checks to test the sensitivity of the main findings are reported in appendix E.

Table 3. Descriptive overview of results for participants with tailored goals (N = 65)

Variable	Condition					
	Control	Reward / Gain frame	Reward / Loss frame	Deposit / Gain frame	Deposit / Loss frame	Total
N	17	20	11	10	7	65
Baseline step count	3406 (1982)	3868 (2673)	4232 (2056)	4036 (3187)	3472 (1537)	3792 (2347)
Assigned step goal	4087 (2378)	4642 (3207)	5078 (2467)	4843 (3825)	4166 (1844)	4550 (2816)
Intervention step count	3130 (2466)	5071 (2783)	4763 (2105)	6395 (4526)	3993 (2464)	4599 (3025)
Days goal achieved	8.00 (5.65)	13.30 (5.49)	10.00 (7.01)	17.40 (6.17)	11.29 (5.16)	11.77 (6.52)

Note: data are means (SD)
"

17a-i) Presentation of process outcomes such as metrics of use and intensity of use

In addition to primary/secondary (clinical) outcomes, the presentation of process outcomes such as metrics of use and intensity of use (dose, exposure) and their operational definitions is critical. This does not only refer to metrics of attrition (13-b) (often a binary variable), but also to more continuous exposure metrics such as “average session length”. These must be accompanied by a technical description how a metric like a “session” is defined (e.g., timeout after idle time) [1] (report under item 6a).

	1	2	3	4	5	
subitem not at all important	○	○	○	◉	○	essential

Selectie wissen

Does your paper address subitem 17a-i?

Copy and paste relevant sections from the manuscript (include quotes in quotation marks "like this" to indicate direct quotes from your manuscript), or elaborate on this item by providing additional information not in the ms, or briefly explain why the item is not applicable/relevant for your study

N/A since in the context of this intervention we did not consider this crucial

17b) For binary outcomes, presentation of both absolute and relative effect sizes is recommended

Does your paper address CONSORT subitem 17b? *

Copy and paste relevant sections from the manuscript (include quotes in quotation marks "like this" to indicate direct quotes from your manuscript), or elaborate on this item by providing additional information not in the ms, or briefly explain why the item is not applicable/relevant for your study

"A Chi-square test of independence showed that requiring a deposit decreased the uptake of the intervention: $\chi 2$ (1, N = 97) = 23.51, p = <.001, V = .492. In the reward conditions 100% of participants accepted the intervention, compared to 61.7% in the deposit conditions (for a descriptive overview of the results see Table 2). "

18) Results of any other analyses performed, including subgroup analyses and adjusted analyses, distinguishing pre-specified from exploratory

Does your paper address CONSORT subitem 18? *

Copy and paste relevant sections from the manuscript (include quotes in quotation marks "like this" to indicate direct quotes from your manuscript), or elaborate on this item by providing additional information not in the ms, or briefly explain why the item is not applicable/relevant for your study

". Due to indications that normality of the DV was violated, we performed a Kruskall-Wallis test to check the robustness of these findings. We only found a significant contrast between the control condition and the deposit/gain condition (p = .001, adjusted with Bonferroni correction). There was no evidence of a significant difference for the other contrasts." and "Furthermore, to test the robustness of these findings, we additionally performed a Kruskall-Wallis test. For the main effects we performed two separate tests, one for each factor from the two-way ANOVA. The interaction effect could not be tested with this alternative method. Consistent with what was found in the two-way ANCOVA, we found that incentive direction was not significantly related to effectiveness (p = .062), but feedback framing was significantly related to effectiveness (p = .033). Additional checks to test the sensitivity of the main findings are reported in appendix E" and "The effect of the manipulations on experienced feelings of loss and goal commitment
To check the effect of our manipulations, we analyzed what the effect was of incentive direction and feedback framing on feelings of loss and goal commitment. We performed two separate two-way ANOVAS (one for feeling of loss, one for goal commitment) with factor incentive direction (deposit/reward) and factor feedback frame (loss/gain). The model included both main effects and their interaction. The first ANOVA with feeling of loss as dependent variable showed a significant effect of incentive direction (F(1, 41) = 19.66, p = <.001, ηp2 = .324). Deposit contracts (M = 7.19, SD = 2.23) resulted in stronger feelings of loss compared to rewards (M = 4.21, SD = 2.19). However, feedback framing did not influence feeling of loss and we did not find a significant interaction. The second ANOVA with goal commitment as dependent variable showed a significant effect of feedback framing (F(1, 41) = 4.95, p = .032, ηp2 = .108). Loss-framed incentives (M = 5.24, SD = 3.11) resulted in weaker goal commitment compared to gain-framed incentives (M = 7.14, SD = 2.37). However, incentive direction did not influence goal commitment and we did not find an interaction."

18-i) Subgroup analysis of comparing only users

A subgroup analysis of comparing only users is not uncommon in ehealth trials, but if done, it must be stressed that this is a self-selected sample and no longer an unbiased sample from a randomized trial (see 16-iii).

	1	2	3	4	5	
subitem not at all important	○	○	○	○	◉	essential

Selectie wissen

Does your paper address subitem 18-i?

Copy and paste relevant sections from the manuscript (include quotes in quotation marks "like this" to indicate direct quotes from your manuscript), or elaborate on this item by providing additional information not in the ms, or briefly explain why the item is not applicable/relevant for your study

Final analyses are reported for users only (who received a tailored goal). "Hypothesis 3-4-5: The effect of incentive direction and feedback framing on effectiveness
A two-way ANCOVA with baseline steps as covariate showed no main effect of incentive direction, F(1, 43) = 1.98, p = .166, ηp2 = .044 indicating that deposits (M = 14.88, SD = 6.40) were not more effective than rewards (M = 12.13, SD = 6.17). We did find a main effect of feedback framing, F(1, 43) = 7.91, p = .007, ηp2 = .155 indicating that loss frames (M = 10.50, SD = 6.22) were significantly less effective than gain frames (M = 14.67, SD = 5.95). Finally, the interaction effect of incentive direction X feedback framing was not significant, F(1, 43) = 1.16, p = .287, ηp2 = .026 indicating that feedback framing did not have a different effect in deposit conditions compared to reward conditions. See Table 3 for a descriptive overview of the results per arm of the experiment.
Furthermore, to test the robustness of these findings, we additionally performed a Kruskall-Wallis test. For the main effects we performed two separate tests, one for each factor from the two-way ANOVA. The interaction effect could not be tested with this alternative method. Consistent with what was found in the two-way ANCOVA, we found that incentive direction was not significantly related to effectiveness (p = .062), but feedback framing was significantly related to effectiveness (p = .033). Additional checks to test the sensitivity of the main findings are reported in appendix E.
"

19) All important harms or unintended effects in each group

(for specific guidance see CONSORT for harms)

Does your paper address CONSORT subitem 19? *

Copy and paste relevant sections from the manuscript (include quotes in quotation marks "like this" to indicate direct quotes from your manuscript), or elaborate on this item by providing additional information not in the ms, or briefly explain why the item is not applicable/relevant for your study

N/A there were no harms or unintended effects

19-i) Include privacy breaches, technical problems

Include privacy breaches, technical problems. This does not only include physical “harm” to participants, but also incidents such as perceived or real privacy breaches [1], technical problems, and other unexpected/unintended incidents. “Unintended effects” also includes unintended positive effects [2].

	1	2	3	4	5	
subitem not at all important	○	○	○	○	◉	essential

Selectie wissen

Does your paper address subitem 19-i?

Copy and paste relevant sections from the manuscript (include quotes in quotation marks "like this" to indicate direct quotes from your manuscript), or elaborate on this item by providing additional information not in the ms, or briefly explain why the item is not applicable/relevant for your study

"Furthermore, a relatively high proportion of participants (36%) did not have historical step data available on their smartphones. These people were assigned a default goal (10.000 steps per day) that in hindsight was unachievable. Future research with a similar goal-setting module should assign more achievable default goals when goals cannot be tailored. In our sample, the mean baseline step count of participants who had historical data was about 3800 steps per day. Based on a meta-analysis of financial incentive intervention effects, we suggest that step goals should not exceed baseline levels by more than 20-30% [15]."

19-ii) Include qualitative feedback from participants or observations from staff/researchers

Include qualitative feedback from participants or observations from staff/researchers, if available, on strengths and shortcomings of the application, especially if they point to unintended/unexpected effects or uses. This includes (if available) reasons for why people did or did not use the application as intended by the developers.

	1	2	3	4	5	
subitem not at all important	○	◉	○	○	○	essential

Selectie wissen

Does your paper address subitem 19-ii?

Copy and paste relevant sections from the manuscript (include quotes in quotation marks "like this" to indicate direct quotes from your manuscript), or elaborate on this item by providing additional information not in the ms, or briefly explain why the item is not applicable/relevant for your study

We did not do this since intervention was fully online

DISCUSSION

22) Interpretation consistent with results, balancing benefits and harms, and considering other relevant evidence

NPT: In addition, take into account the choice of the comparator, lack of or partial blinding, and unequal expertise of care providers or centers in each group

22-i) Restate study questions and summarize the answers suggested by the data, starting with primary outcomes and process outcomes (use)

Restate study questions and summarize the answers suggested by the data, starting with primary outcomes and process outcomes (use).

	1	2	3	4	5	
subitem not at all important	○	○	○	○	◉	essential

Selectie wissen

Does your paper address subitem 22-i? *

Copy and paste relevant sections from the manuscript (include quotes in quotation marks "like this" to indicate direct quotes from your manuscript), or elaborate on this item by providing additional information not in the ms, or briefly explain why the item is not applicable/relevant for your study

"This study found that financial incentives increase intervention effects compared to an active no incentive control condition. Furthermore, as expected, results show that self-funded deposit contracts for physical activity have a lower uptake than regular reward incentives. Yet, contrary to our hypothesis, we did not find deposit contracts to be more effective than reward incentives. An important unexpected finding was that loss framing decreased effectiveness of the intervention compared to gain framing. "

22-ii) Highlight unanswered new questions, suggest future research

Highlight unanswered new questions, suggest future research.

	1	2	3	4	5	
subitem not at all important	○	○	○	○	◉	essential

Selectie wissen

Does your paper address subitem 22-ii?

Copy and paste relevant sections from the manuscript (include quotes in quotation marks "like this" to indicate direct quotes from your manuscript), or elaborate on this item by providing additional information not in the ms, or briefly explain why the item is not applicable/relevant for your study

"Our results contradict the findings by Patel et al. [21] who showed that loss-framed incentives were more effective than gain-framed incentives. However, Patel et al. [21] studied obese university employees with a BMI higher than 27, whereas our sample consisted of healthy university students. Possibly, a difference in regulatory fit – related to differences in study sample - might explain this discrepancy. Regulatory fit is when the persuasiveness of a health message is increased when its frame is congruent with the regulatory orientation of the individual [56]. Regulatory focus theory discerns two modes of regulatory orientation: promotion focus and prevention focus. Where people with a promotion focus aim for desired end states, people with a prevention focus aim for avoiding undesired end states [56]. Perhaps, obese adults are more focused on avoiding obesity-related health problems and therefore have a stronger prevention focus when increasing physical activity. This could then lead them to respond better to a loss-framed incentive (in which losing money is prevented) because of a greater experienced regulatory fit. On the other hand, perhaps healthy students have a stronger promotion focus (on becoming more fit rather than avoiding health problems) and therefore respond better to a gain-framed incentive. Whether the regulatory fit effect also applies to incentive framing (and not only to framing of persuasive health messages) is an interesting avenue for future research. Future research should measure regulatory orientation and investigate possible interactions with different incentive frames. "

20) Trial limitations, addressing sources of potential bias, imprecision, and, if relevant, multiplicity of analyses

20-i) Typical limitations in ehealth trials

Typical limitations in ehealth trials: Participants in ehealth trials are rarely blinded. Ehealth trials often look at a multiplicity of outcomes, increasing risk for a Type I error. Discuss biases due to non-use of the intervention/usability issues, biases through informed consent procedures, unexpected events.

	1	2	3	4	5	
subitem not at all important	○	○	○	○	◉	essential

Selectie wissen

Does your paper address subitem 20-i? *

Copy and paste relevant sections from the manuscript (include quotes in quotation marks "like this" to indicate direct quotes from your manuscript), or elaborate on this item by providing additional information not in the ms, or briefly explain why the item is not applicable/relevant for your study

"However, requiring a deposit beforehand also resulted in lower uptake of the deposit contract conditions. As a result, the deposit requirement may have filtered out the people that were lacking motivation, thus leading to an overestimation of effectiveness in the deposit contract conditions. Consequently, caution is warranted when interpreting the effectiveness of the deposit contract conditions. Another limitation of our study is that high dropout before onboarding, unbalanced allocation, lack of uptake in the deposit contract conditions, and the exclusion of non-tailored goals decreased the statistical power of our analyses. Furthermore, before onboarding, participants read the informed consent which mentioned that the study possibly required them to deposit € 10 of their own money. Mentioning this possibility was important for informed consent, but may have deterred some participants from participating before they onboarded in the application. It is possible that this biased our analysis of uptake, and that the actual uptake of deposit contracts is lower than our analyses suggest. Additionally, although we propose that objective measures of physical activity are superior to subjective self-reports, an important criticism of pedometer-based intervention research is that it is impossible to differentiate an increase in step count from an increase in pedometer wear time [57]. In our case, especially participants in the gain-framed conditions indeed reported to have carried their smartphone more often than they normally do (see appendix E), and this might partly explain why gain-framed conditions were more effective than loss-framed conditions. Furthermore, a relatively high proportion of participants (36%) did not have historical step data available on their smartphones. These people were assigned a default goal (10.000 steps per day) that in hindsight was unachievable. Future research with a similar goal-setting module should assign more achievable default goals when goals cannot be tailored. In our sample, the mean baseline step count of participants who had historical data was about 3800 steps per day. Based on a meta-analysis of financial incentive intervention effects, we suggest that step goals should not exceed baseline levels by more than 20-30% [15]. Additionally, the intervention was launched in March 2020 and in this period the first COVID-19 lockdown measures in the Netherlands were implemented. Although this probably impacted all conditions equally, a large part (78.5%) of the sample indeed reported having been less physically active than they normally are due to the situation around COVID-19. As a result, it is possible that estimates of baseline activity were lower than normal and therefore the intervention led to stronger improvements than would be found under normal circumstances. "

21) Generalisability (external validity, applicability) of the trial findings

NPT: External validity of the trial findings according to the intervention, comparators, patients, and care providers or centers involved in the trial

21-i) Generalizability to other populations

Generalizability to other populations: In particular, discuss generalizability to a general Internet population, outside of a RCT setting, and general patient population, including applicability of the study results for other organizations

	1	2	3	4	5	
subitem not at all important	○	○	○	○	◉	essential

Selectie wissen

Does your paper address subitem 21-i?

Copy and paste relevant sections from the manuscript (include quotes in quotation marks "like this" to indicate direct quotes from your manuscript), or elaborate on this item by providing additional information not in the ms, or briefly explain why the item is not applicable/relevant for your study

"Furthermore, our sample consisted of predominantly healthy, young, female students at universities. Although we purposefully recruited a homogenous sample to increase internal validity, the external validity of our findings is therefore restricted. Older or more chronically ill populations might respond differently to this type of intervention. Finally, we only investigated short-term effects during a 20-day intervention period. Therefore, we are unable to answer questions about the long-term effectiveness of the different incentive directions and incentive frames that we tested."

21-ii) Discuss if there were elements in the RCT that would be different in a routine application setting

Discuss if there were elements in the RCT that would be different in a routine application setting (e.g., prompts/reminders, more human involvement, training sessions or other co-interventions) and what impact the omission of these elements could have on use, adoption, or outcomes if the intervention is applied outside of a RCT setting.

	1	2	3	4	5	
subitem not at all important	○	○	◉	○	○	essential

Selectie wissen

Does your paper address subitem 21-ii?

Copy and paste relevant sections from the manuscript (include quotes in quotation marks "like this" to indicate direct quotes from your manuscript), or elaborate on this item by providing additional information not in the ms, or briefly explain why the item is not applicable/relevant for your study

This was not addressed specifically

OTHER INFORMATION

23) Registration number and name of trial registry

Does your paper address CONSORT subitem 23? *

Copy and paste relevant sections from the manuscript (include quotes in quotation marks "like this" to indicate direct quotes from your manuscript), or elaborate on this item by providing additional information not in the ms, or briefly explain why the item is not applicable/relevant for your study

OSF Registries, https://osf.io/34ygt

24) Where the full trial protocol can be accessed, if available

Does your paper address CONSORT subitem 24? *

Cite a Multimedia Appendix, other reference, or copy and paste relevant sections from the manuscript (include quotes in quotation marks "like this" to indicate direct quotes from your manuscript), or elaborate on this item by providing additional information not in the ms, or briefly explain why the item is not applicable/relevant for your study

This was not published prior to performing the study

25) Sources of funding and other support (such as supply of drugs), role of funders

Does your paper address CONSORT subitem 25? *

Copy and paste relevant sections from the manuscript (include quotes in quotation marks "like this" to indicate direct quotes from your manuscript), or elaborate on this item by providing additional information not in the ms, or briefly explain why the item is not applicable/relevant for your study

"Acknowledgements
This work was supported by The Netherlands Cardiovascular Research Initiative: an initiative with support of the Dutch Heart Foundation, CVON2016-12 BENEFIT, ZonMw (The Netherlands Organization for Health Research and Development), Leiden University and the members of the BENEFIT consortium. Furthermore, we would like to thank Fiona Brosig (FB; Research Master student Psychology, Leiden University) for her assistance in developing the intervention, recruiting participants and running the experiment."

X27) Conflicts of Interest (not a CONSORT item)

X27-i) State the relation of the study team towards the system being evaluated

In addition to the usual declaration of interests (financial or otherwise), also state the relation of the study team towards the system being evaluated, i.e., state if the authors/evaluators are distinct from or identical with the developers/sponsors of the intervention.

	1	2	3	4	5	
subitem not at all important	○	○	○	○	◉	essential

Selectie wissen

Does your paper address subitem X27-i?

Copy and paste relevant sections from the manuscript (include quotes in quotation marks "like this" to indicate direct quotes from your manuscript), or elaborate on this item by providing additional information not in the ms, or briefly explain why the item is not applicable/relevant for your study

"Conflicts of Interest
PS and TK are affiliated with the Centre for Digital Health Interventions, a joint initiative of the Department of Management, Technology, and Economics at ETH Zurich and the Institute of Technology Management at the University of St Gallen, which is funded in part by the Swiss health insurer, CSS. TK is also cofounder of Pathmate Technologies, a university spin-off company that creates and delivers digital clinical pathways. However, neither CSS nor Pathmate Technologies was involved in this study.
"

About the CONSORT EHEALTH checklist

As a result of using this checklist, did you make changes in your manuscript? *

- ○ yes, major changes
- ○ yes, minor changes
- ◉ no

What were the most important changes you made as a result of using this checklist?

Jouw antwoord

How much time did you spend on going through the checklist INCLUDING making changes in your manuscript *

90 minutes in total time spent

As a result of using this checklist, do you think your manuscript has improved? *

- ○ yes
- ◉ no
- ○ Anders:

Would you like to become involved in the CONSORT EHEALTH group?

This would involve for example becoming involved in participating in a workshop and writing an "Explanation and Elaboration" document

- ○ yes
- ◉ no
- ○ Anders:

Selectie wissen

Any other comments or questions on CONSORT EHEALTH

Jouw antwoord

STOP - Save this form as PDF before you click submit

To generate a record that you filled in this form, we recommend to generate a PDF of this page (on a Mac, simply select "print" and then select "print as PDF") before you submit it.

When you submit your (revised) paper to JMIR, please upload the PDF as supplementary file.

Don't worry if some text in the textboxes is cut off, as we still have the complete information in our database. Thank you!

Final step: Click submit !

Click submit so we have your answers in our database!

Verzenden Formulier wissen

Verzend nooit wachtwoorden via Google Formulieren.

Deze content is niet gemaakt of goedgekeurd door Google. Misbruik rapporteren - Servicevoorwaarden - Privacybeleid

Google Formulieren
